# Microglial VRK2 Regulates Astrocytic GABA Synthesis and Tonic Inhibition in the Thalamus

**DOI:** 10.1002/glia.70101

**Published:** 2025-11-20

**Authors:** Dongsu Lee, Go Eun Ha, Yeleen Lee, Denise Lee, Jongseo Lee, Jae Ho Yoon, Leechung Chang, Kyung Won Jo, Ho‐Keun Kwon, Kyong‐Tai Kim, Eunji Cheong

**Affiliations:** ^1^ Department of Biotechnology, College of Life Science and Biotechnology Yonsei University Seoul Republic of Korea; ^2^ Department of Microbiology and Immunology Yonsei University College of Medicine Seoul Republic of Korea; ^3^ Laboratory of Molecular Neurophysiology, Department of Life Sciences Pohang University of Science and Technology (POSTECH) Pohang Gyeongbuk Republic of Korea; ^4^ Hesed Bio Corporation Pohang Gyeongbuk Republic of Korea; ^5^ Institute for Immunology and Immunological Diseases, Yonsei University College of Medicine Seoul Republic of Korea; ^6^ Brain Korea 21 PLUS Project for Medical Sciences, Yonsei University College of Medicine Seoul Republic of Korea; ^7^ Generative Genomics Research Center, Global Green Research & Development Center Handong Global University Pohang Republic of Korea

**Keywords:** microglia, microglia‐astrocyte signaling, neurodevelopment disorder, thalamus, tonic GABA synthesis, tonic inhibition, vaccinia‐related kinase 2

## Abstract

Vaccinia‐related kinase 2 (VRK2) is a prominent genetic risk factor for neurodevelopmental disorders (NDDs), including schizophrenia and epilepsy, which are characterized by cognitive and behavioral impairments. The mediodorsal (MD) thalamus, a higher‐order nucleus involved in executive function and social behavior, is frequently disrupted in these conditions. However, how VRK2 influences thalamic regulation remains unclear. Here, we show that *Vrk2*‐deficient mice exhibit a significant reduction in tonic GABA currents in the MD thalamus, accompanied by decreased excitatory synaptic input but preserved intrinsic neuronal excitability. Although VRK2 is not expressed in astrocytes, its deletion impaired astrocyte‐mediated tonic inhibition, suggesting a non‐cell‐autonomous mechanism. Single‐cell and bulk transcriptomic analyses revealed that VRK2 is specifically expressed in microglia and that its loss alters cytokine signaling pathways. Pharmacological depletion of microglia or TNF‐α inhibition in wild‐type mice recapitulated the tonic inhibition deficits observed in *Vrk2*‐deficient animals. Further, astrocyte‐specific interventions revealed that tonic GABA is synthesized through the DAO–ALDH1A1 pathway, which was selectively downregulated in the absence of VRK2, while MAOB, BEST1, and GABA receptor components remained unchanged. These findings define a novel glial–glial signaling axis in which microglial VRK2 maintains thalamic inhibitory tone through cytokine‐dependent regulation of astrocytic GABA synthesis. This mechanism operates across both first‐ and higher‐order thalamic nuclei and may underlie sensory and cognitive impairments associated with VRK2‐linked NDDs. Our work provides new insight into glial coordination as a critical regulator of tonic inhibition and highlights microglial cytokine signaling as a molecular bridge between genetic risk and circuit‐level dysfunction.

## Graphical abstract

- In *Vrk2*‐deficient mice, tonic GABA inhibition is reduced in the mediodorsal thalamus due to impaired astrocytic GABA synthesis.
- Microglial VRK2 regulates this process via TNF‐α signaling, revealing a glial–glial mechanism of thalamic inhibition.

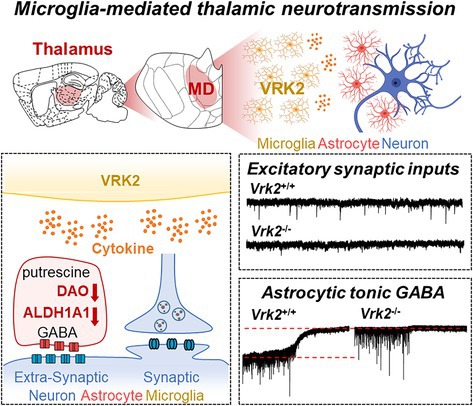

## 1 Introduction

Neurodevelopmental disorders (NDDs), including schizophrenia, autism spectrum disorder (ASD), and intellectual disability, represent a major public health challenge, with substantial societal and personal burdens. Despite decades of research, the underlying molecular and circuit mechanisms remain poorly understood, and current treatments are largely symptomatic. To develop targeted interventions, there is an urgent need to uncover the genetic and cellular mechanisms that contribute to early brain vulnerability and long‐term cognitive dysfunction.

Among the risk genes implicated in NDDs, *Vaccinia‐related kinase 2 (Vrk2)* has emerged as a recurrent genetic factor. VRK2, encoded by the *Vrk2* gene, has been repeatedly associated with schizophrenia and related NDDs in genome‐wide association studies (GWAS) (Consortium et al. 2012; Lee et al. 2019; Li, Wang, et al. 2012; Steinberg et al. 2011; Zhang et al. 2015). Notably, common risk variants of *Vrk2*, such as rs2312147 and rs3732136 are associated with altered cortical and subcortical brain function in schizophrenia, while rs1302641 has been linked to epilepsy (Lee et al. 2019). Despite these robust genetic associations, the precise cellular and circuit mechanisms by which VRK2 contributes to disease phenotypes remain unclear.

*Vrk2*‐deficient mice display a spectrum of behavioral abnormalities—including deficits in social interaction, fear memory, and spatial memory—that recapitulate key features of schizophrenia and ASD (Consortium et al. 2012; Lee et al. 2019; Li, Wang, et al. 2012; Steinberg et al. 2011; Zhang et al. 2015). These behavioral domains are supported by multiple interconnected brain regions—including the medial prefrontal cortex, hippocampus, amygdala, and thalamus (Griffin 2015; Mitchell 2015; Rubin et al. 2014; Silva et al. 2016; Zelikowsky et al. 2014). While prior studies emphasized hippocampal circuit dysfunction in *Vrk2*‐deficient mice (Lee et al. 2019), accumulating evidence implicates dysfunction in high‐order thalamocortical circuits—especially those involving the mediodorsal (MD) thalamus—in working memory and executive function (Anastasiades et al. 2021; Collins et al. 2018; Fang et al. 2025; Ferguson and Gao 2018; Karimi et al. 2021; Lee et al. 2011; Paydar et al. 2014; Zhou et al. 2017).

The MD thalamus, in particular, serves as a critical hub that integrates afferents from the medial prefrontal cortex, hippocampus, and amygdala and supports top‐down modulation of cognitive functions such as social behavior, social hierarchy, and behavioral flexibility (Anastasiades et al. 2021; Collins et al. 2018; Ferguson and Gao 2018; Karimi et al. 2021; Lee et al. 2011; Paydar et al. 2014; Zhou et al. 2017). Human neuroimaging studies consistently report structural and functional abnormalities in the thalamus—including the MD nucleus—in individuals with schizophrenia, ASD, and elevated clinical risk (Anticevic et al. 2015; Chen et al. 2019; Giraldo‐Chica et al. 2018; Nair et al. 2013; Pergola et al. 2015; Woodward et al. 2012). Altered MD thalamic connectivity has been directly linked to executive dysfunction and impaired cognitive control (Anticevic et al. 2014; Giraldo‐Chica et al. 2018), making it a strong candidate for mediating VRK2‐associated phenotypes.

While traditionally studied from a neuronal perspective, thalamic function is increasingly recognized as being shaped by non‐neuronal cells. In particular, astrocytes in the thalamus release GABA tonically, generating a persistent inhibitory tone that modulates thalamocortical excitability via activation of extra‐synaptic GABA_A_ receptors (Koh et al. 2023; Kwak et al. 2020; Paydar et al. 2014). This astrocyte‐mediated tonic inhibition modulates thalamocortical excitability and cognitive flexibility, with pharmacological modulation of extra‐synaptic GABA_A_ receptors in the MD thalamus altering fear extinction, underscoring its behavioral relevance (Paydar et al. 2014).

Importantly, *Vrk2* expression is highly enriched in microglia but absent from astrocytes and neurons. Quantitative PCR analyses with single‐cell sorting by MACS have shown that *Vrk2* is predominantly expressed in CD11b^+^ cells, putatively IBA1^+^ microglia in multiple brain regions, including the hippocampus and cortex, but not in REELIN^+^, CAMKII^+^, or GFAP^+^ populations (Lee et al. 2019). In the same study, immunohistochemistry analysis also shows that LacZ proteins are expressed in the IBA1^+^ microglia, within the hippocampus of transgenic mice, in which *Vrk2* is associated with *LacZ* (Lee et al. 2019). This microglial specificity positions VRK2 as a potential regulator of microglia‐dependent circuit functions.

Notably, the microglia are increasingly recognized as active participants in NDD pathology. Transcriptomic studies in ASD have shown dysregulation of microglial genes (e.g., *Fyb*, *Syk*, *Foxp2*, *Auts2*) and region‐specific alterations in microglial density and activation state (Li and Barres 2018; Matuleviciute et al. 2023). Microglial morphological priming, increased MHC‐II expression, and upregulation of pro‐inflammatory miRNAs (e.g., miR‐155) have been observed in ASD‐affected cortical and limbic regions (Matuleviciute et al. 2023). These findings point to altered microglial signaling as a central feature of disease.

Traditionally, microglia have been recognized for their roles in neuroinflammation and innate immune responses (Li and Barres 2018). However, beyond inflammation, microglia contribute to developmental and adult circuit remodeling (Li and Barres 2018). They regulate neuronal activity, prune immature excitatory synapses, and respond dynamically to changes in local circuit activity, even in adulthood (Li and Barres 2018). Microglia preferentially interact with highly active neurons, often those with elevated intracellular calcium, and can attenuate this activity through direct contact (Li, Du, et al. 2012). During early postnatal development, microglia modulate synapses depending on P2RY12 and CX3CR1 signaling pathways, and prune vGLUT2^+^ presynaptic terminals in the dorsolateral geniculate nucleus (dLGN) (Li and Barres 2018). Microglia also target C1q and C3‐tagged synapses for elimination via CR3, as demonstrated by the excessive excitatory synapses in C1q‐deficient cortex (Li and Barres 2018). Imaging studies further show that microglial synaptic pruning preferentially affects excitatory synapses during critical periods (Lee et al. 2021), though context‐dependent effects on inhibitory synapses have also been observed. Given that *Vrk2* is specifically expressed in microglia, its loss impairs this pruning process, suppressing synaptosome engulfment in vitro, increasing immature excitatory synapses, and reducing excitatory transmission in the hippocampus (Lee et al. 2019). These findings position microglia not merely as immune sentinels but as central regulators of circuit remodeling and stability across development and into adulthood.

One key mechanism by which microglia influence circuit function is through the release of cytokines. In both injury and disease, microglia release cytokines such as IL‐1β and TNF‐α, which modulate astrocytic reactivity and gliotransmitter release (Jha et al. 2019; Matejuk and Ransohoff 2020). While extensively studied in pathological contexts, microglia–astrocyte crosstalk under homeostatic or NDD‐relevant conditions remains less well understood.

These insights raise the possibility that VRK2 may influence thalamic circuit function through multiple glial mechanisms. Based on prior evidence, we hypothesized that microglia modulate thalamic activity not only via synaptic pruning but also through interactions with astrocytes that affect neuronal excitability (Koh et al. 2023; Kwak et al. 2020). Such cross‐cellular signaling may play a critical role in regulating astrocytic GABA release, thereby modulating the extracellular inhibitory tone and shaping thalamocortical dynamics.

In this study, we investigated how VRK2 regulates the function of the MD thalamus. After identifying the robust thalamic expression of *Vrk2*, we characterized the physiological properties of MD thalamocortical neurons, including neuronal intrinsic excitability, synaptic transmission, and tonic inhibition. Given the microglia‐specific expression of *Vrk2*, we further examined how its deletion affects microglial abundance, morphology, and cytokine signaling, and how these changes impact astrocytic GABA synthesis. By dissecting this microglia–astrocyte–neuron axis, our findings aim to reveal a novel glial‐mediated mechanism by which VRK2 regulates thalamic circuit function, providing new insight into the cellular basis of attention and cognitive impairments in VRK2‐associated NDDs.

## 2 Methods

### 2.1 Animals

*Vrk2*‐deficient mice were generated by crossing VRK2^tm1a (KOMP)Wtsi^ mice (CSD41170) from the UC Davis Knockout Mouse Project (KOMP) with protamine‐Flp and protamine‐Cre transgenic mice (Lee et al. 2019). The final *Vrk2*‐deficient mice were provided by Kim's laboratory (POSTECH, Pohang, Korea) (Lee et al. 2019). Wild‐type (C57BL/6J) and *Vrk2*‐deficient mice were maintained under a 12:12‐h light–dark cycle (lights on at 7:00 A.M.) and had *ad libitum* access to food and water. Animal care and handling were performed following the guidelines of the Institutional Animal Care and Use Committee at Yonsei University (Seoul, Korea). Animal experiments were approved by the Institutional Animal Care and Use Committee of Korea (approval number: IACUC‐A‐202503‐2014‐02). To generate microglia‐depleted mice (Ki20227‐administered mice), colony‐stimulating factor 1 receptor kinase inhibitors (Ki20227, 250 μg/mL in water; orb181032, Biorbyt, Cambridge, UK) are administered to the 4–5‐week‐old male C57BL/6J mice with a drink for seven consecutive days (Yamamoto et al. 2019). To generate TNF‐α‐inhibited mice (C87‐injected mice), stereotaxic injections were performed. 0.6 μL of 1 M phosphate‐buffered saline (PBS; 137 mM NaCl, 2.7 mM KCl, 1.5 mM KH_2_PO_4_, 10 mM Na_2_HPO_4_; pH 7.2–7.4) or 1 M PBS with 4 mM C87 (5484, Tocris Bioscience, Bristol, UK) was injected into the thalamus (anteroposterior: −1.7 mm, mediolateral: ±1.6 mm, dorsoventral: −3.4 mm from cortical surface), a TNF‐α inhibitor. All experiments were conducted 2 h after injection (Yamamoto et al. 2019).

### 2.2 Single‐Cell RNA Sequencing

For thalamic RNA sequencing, all procedures and analyses were conducted according to the manufacturer's protocol (Macrogen, Seoul, Korea). The thalamus was isolated from the 11‐week male wild‐type (C57BL/6J) mice (3 mice). Single‐cell RNA sequencing was performed using the 10× Genomics Chromium Single Cell 3′ Reagent Kit v2, followed by sequencing on the Illumina HiSeq X platform. A single‐cell suspension was processed to generate barcoded cDNA libraries, in which mRNA transcripts from individual cells were reverse‐transcribed and labeled with unique cell barcodes and UMIs. The resulting libraries were sequenced in a paired‐end format with the read configuration of 26 bp for Read 1 (cell barcode and UMI), 8 bp for the i7 index read, and 98 bp for Read 2 (cDNA fragment). For the initial processing, sequencing data were processed using the Cell Ranger pipeline (v2.2.0, RRID: SCR_017344). The pipeline begins with cellranger mkfastq, which serves as a wrapper for Illumina's bcl2fastq software. This step demultiplexes raw base call (BCL) files into FASTQ format and separates reads by sample indices, while also preserving barcode and UMI information essential for single‐cell resolution. The data were clustered using the Seurat R Package. The raw UMI count matrix was first imported and converted into Seurat objects. Cells were filtered based on quality control metrics such as the number of detected genes, total counts, and mitochondrial gene content. The data (7872 cells) were log‐normalized, and highly variable features were identified to focus downstream analyses. These features were scaled and used for PCA, which served as the input for SNN graph‐based clustering. Dimensionality reduction was performed using UMAP to visualize the identified clusters. Differentially expressed genes (DEGs) were identified for each cluster using the Wilcoxon rank‐sum test (|fold change| > 2 and *p*‐value < 0.05) to determine cluster‐specific markers. These markers were used to annotate major cell types and interpret transcriptional differences between groups.

### 2.3 Bulk‐RNA Sequencing

All procedures and analyses were conducted according to the manufacturer's protocol (Macrogen, Korea). The thalamus was isolated from 4 to 6 weeks old wild‐type (C57BL/6J) and *Vrk2*‐deficient mice (three mice in each group). Then, the thalamus samples underwent paired‐end RNA sequencing using the TruSeq Stranded mRNA LT sample Prep Kit, followed by sequencing on the Illumina platform. The total RNA was isolated from the thalamus samples, purified using an RNA purification kit after eliminating DNA contamination by DNase, and randomly fragmented. The RNA fragments were turned into cDNA by reverse transcription and ligation. After emphasizing the cDNA into a 200–400 bp insert size. Then, the cDNA fragment underwent paired‐end sequencing.

Every raw read underwent quality control (QC) analysis based on total bases, total reads, GC%, etc. After eliminating artifacts such as adaptor sequence using Trimmomatic (RRID: SCR_011848), contaminant DNA, and PCR duplicates, the trimmed reads were aligned with the reference genome using HISAT2 (RRID: SCR_015530) and underwent transcript assembly using StringTie (RRID: SCR_016323). The results of transcript quantification were calculated into expression profiles: Fragments Per Kilobase of transcript per Million mapped reads (FPKM), Reads Per Kilobase of transcript per Million mapped reads (RPKM), and Transcripts Per Kilobase Million (TPM). For data quality check, we remove the gene expression that has 0 count in any samples (24,715 reads are removed from 45,777 reads, 21,062 reads left). Then, gene expressions underwent relative log expression (RLE) normalization, fold change calculation, and negative binomial Wald test (nbinomWaldTest) to reduce the systematic bias by size factor using the DEseq2 R library (RRID: SCR_000154). The DEGs (270 genes) were selected by the Wilcoxon rank sum test (|fold change| > 1.5 and *p*‐value < 0.05) of expression values. Functional annotation and gene‐set enrichment analysis were performed on DEGs, based on the KEGG database (https://www.genome.jp/kegg/).

To compare the DEGs in the thalamus of *Vrk2*‐deficient mice with DEGs in the microglia‐depleted mice. We adopted the RNA sequencing database (https://rnaseq.mind.uci.edu/green/ad_plx/) from a published previous study (Spangenberg et al. 2019) using dissected thalamus striatum tissues from wild‐type mice with vehicle or PLX5622. We selected DEGs by the same standard as RNA sequencing in the *Vrk2*‐deficient mice (|fold change| > 1.5 and *p*‐value < 0.05) and compared the selected list of genes with DEGs in the *Vrk2*‐deficient mice.

### 2.4 Cell Sorting

For cell sorting, the whole thalamus from the left and right hemispheres of the 4–6 week C57BL/6 mice was dissected (three samples, five mice for each sample). The brain tissues were mechanically minced using an adult brain dissociation kit (mouse and rat; 130‐107‐677, Miltenyi Biotec, Bergisch Gladbach, Germany). The digested brain tissue was filtered through a 70‐μm strainer and centrifuged at 300 rcf for 10 min at a temperature of 4°C. Pellets were resuspended with Hank's balanced salt solution (HBSS). Then, debris was removed by the debris removal solution in the dissociation kit, and red blood cells were removed by the 1× red blood cell removal solution in the dissociation kit. The digested brain tissues were centrifuged at 300 rcf for 5 min at a temperature of 4°C. The pellets were resuspended in a 0.5% bovine serum albumin (BSA) solution for further analysis.

For cell sorting, isolated cells were blocked with TruStain FcX PLUS (BioLegend, San Diego, CA, USA) and stained with anti‐CD11B‐PE‐Cy7 (1:200; clone M1/70, 101216, BioLegend, San Diego, CA, USA) and anti‐CD45‐BV605 (1:200; clone 30‐F11, 103140, BioLegend, San Diego, CA, USA) in PBS. After staining, cells were washed with PBS, resuspended, and DAPI was added immediately before sorting to exclude non‐viable cells. Cell sorting was performed on a Sony MA900 (Sony, Tokyo, Japan). Microglia were defined as CD11bmidCD45mid among singlet, DAPI− events, and CD11b−CD45− cells were collected as a non‐microglia fraction. Post‐sort purity was assessed in each experiment using a small aliquot of the stained sample and was routinely > 90%. For RNA extraction, cells were sorted directly into Buffer RLT (Qiagen, Hilden, Germany) supplemented with β‐mercaptoethanol, and the total RNA was isolated using the RNeasy Micro Kit (Qiagen, Hilden, Germany) according to the manufacturer's instructions. The gating strategy for cell sorting is shown in Figure S1. RNA (30 ng) for each sample was reverse‐transcribed using SuperScript IV Reverse Transcriptase (18,090,010, Thermo Fisher Scientific, Waltham, MA, USA) to generate cDNA.

### 2.5 Quantitative Polymerase Chain Reaction

**FIGURE 1:**
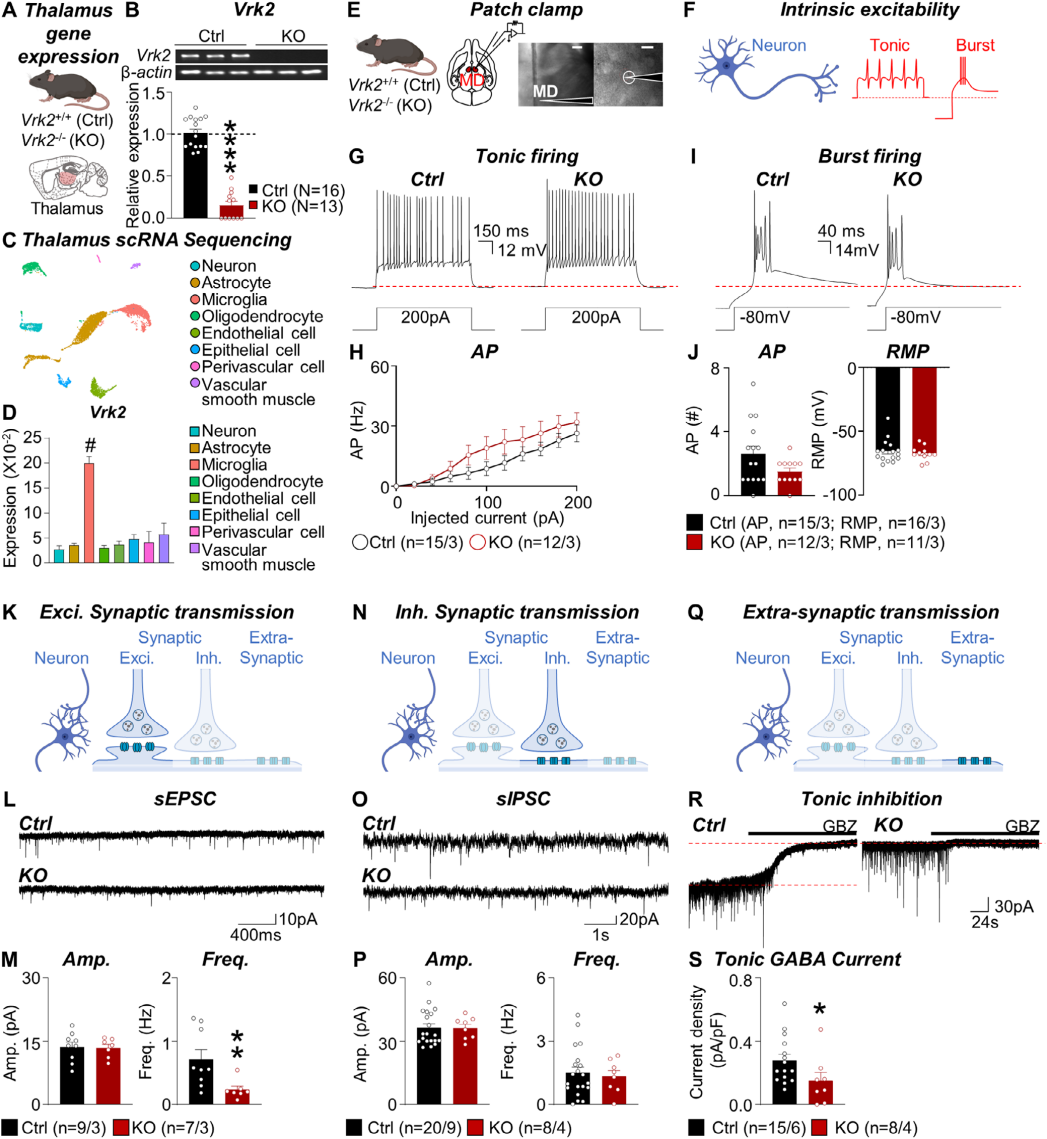
Molecular profiling and electrophysiological assessment of the mediodorsal thalamus in *Vrk2*‐deficient mice. (A) Schematic of quantitative PCR (qPCR) analysis of the thalamus in the control (*Vrk2* ^+/+^, Ctrl) and *Vrk2*‐deficient (*Vrk2* ^−/−^, KO) mice. (B) PCR analysis of *Vrk2* in the thalamus of three representative Ctrl and KO mice (*top*). Quantification of *Vrk2* expression in the thalamus of Ctrl and KO mice (*bottom*). (C) The single‐cell RNA (scRNA) sequencing clusters of the thalamus, annotated with cell types: neuron, astrocyte, microglia, oligodendrocyte, endothelial cell, epithelial cell, perivascular cell, and vascular smooth muscle. (D) Quantification of *Vrk2* expression in the thalamus cell types. (E) Schematics (*left*) and representative images (*right*) of ex vivo electrophysiology in the mediodorsal (MD) thalamus of Ctrl and KO mice. Scale bar, 200 and 20 μm. (F) Schematic of intrinsic excitability in the MD thalamus. (G) Representative trace for tonic firing in Ctrl and KO mice. (H) Quantification of the number of action potentials (AP) in 1‐s steps, per injected current from 0 to 200 pA. (I) Representative trace for burst firing in Ctrl and KO mice. (J) Quantification of the number of action potentials (AP) in a burst and resting membrane potential (RMP). (K) Schematic of excitatory synaptic transmission in the thalamus. (L) Representative traces for excitatory postsynaptic currents (EPSCs) in Ctrl and KO mice. (M) Quantification of amplitude (Amp.) and frequency (Freq.) of the EPSCs in the Ctrl and KO mice. (N) Schematic of inhibitory synaptic transmission in the thalamus. (O) Representative traces for inhibitory postsynaptic currents (IPSCs) in Ctrl and KO mice. (P) Quantification of Amp. and Freq. of the IPSCs in the Ctrl and KO mice. (Q) Schematic of extra‐synaptic transmission in the thalamus. (R) Representative trace of tonic inhibition in the MD thalamus in Ctrl and KO mice. (S) Quantification of tonic GABA current in the MD thalamus in Ctrl and KO mice. ‘*N*’ denotes the number of mice (B). ‘*n*’ denotes the number of cells/mice (H, J, M, P, and S). Data are presented as mean ± SEM (B, D, H, J, M, P, and S). ‘#’ denotes a differentially expressed gene (DEG) (D). *p < 0.05, **p < 0.01, and ****p < 0.0001; Mann–Whitney test (B, J, M, P, and S), two‐way ANOVA with Sidak's post hoc test (H). See also Figures S1 and S2.

RNA was extracted from the thalamus of 4–6 weeks‐old C57BL/6 mice (for Figures 1 and 4), 4–6 weeks‐old *Vrk2*‐deficient mice (for Figures 1 and 4), 4–6 weeks‐old C87‐injected mice (for Figure 4), and 1–14 weeks‐old C57BL/6 mice (for Figure S1) using TRIzol reagent (15596026, Invitrogen, Carlsbad, CA, USA). RNA (1 μg) for each sample was reverse‐transcribed using SuperScript III Reverse Transcriptase (18,080,051, Thermo Fisher Scientific, Waltham, MA, USA) to generate cDNA. To examine the expression of *Vrk2* in the sorted thalamic microglia.

cDNA from the mouse thalamus was used as a template for PCR analysis. The primers were: *Vrk2‐*F, 5′‐TGC AAG ACA TGT CAT AAA GCT G‐3′ (Lee et al. 2019); *Vrk2‐*R, 5′‐TAG TCT GCA AGA TAA ACC CG‐3′ (Lee et al. 2019); *Dao‐*F, 5′‐GGA GGC CCC TTG GAT TAA ACA‐3′; *Dao‐*R, 5′‐TGG TAT TGT GGT CAC GGA CG‐3′; *Aldh1a1‐*F, 5′‐CAA GGC CAA TGT TGT GTC GC‐3′; *Aldh1a1‐*R, 5′‐CTG GGG TCA GAG GAT TTC CA‐3′; *Maob‐*F, 5′‐CTC TCG TGT GCC TTT GGG TT‐3′; *Maob‐*R, 5′‐GCA TAG GTG CCA TCT GGT TTG‐3′; *Best1‐*F, 5′‐GGT GTC TAG CTT CGT GGA GG‐3′; *Best1‐*R, 5′‐AAA GCG CTT GTA GAC CGA GG‐3′; *Gabrd‐*F, 5′‐GTG GCC AGC ATT GAC CAT ATC‐3′; *Gabrd‐*R, 5′‐TCT GAT GCA GGA ACA CAG TCA TG‐3′; *mouse β‐actin*‐F, 5′‐AGC AAG CAG GAG TAC GAT GAG‐3′; and *mouse β‐actin‐*R, 5′‐AGC TCA GTA ACA GTC CGC CT‐3′; *Aldh1l1*‐F, 5′‐ATG ATC ATC TCT CGG TTT GCT GA‐3′ (Bravo‐Ferrer et al. 2022); *Aldh1l1*‐R, 5′‐CAT CGG TCT TGT TGT ATG TGT TG‐3′ (Bravo‐Ferrer et al. 2022); *Rbfox3*‐F, 5′‐GCG GTC GTG TAT CAG GAT GG‐3′ (Bravo‐Ferrer et al. 2022); *Rbfox3*‐R, 5′‐CGA TGC TGT AGG TTG CTG TG‐3′ (Bravo‐Ferrer et al. 2022); *Aif1*‐F, 5′‐TCC CCC AGC CAA GAA AGC TA‐3′ (Bravo‐Ferrer et al. 2022); *Aif1*‐R, 5′‐GAT GTG ACC CAC TAG GAG CG‐3′ (Bravo‐Ferrer et al. 2022).

To investigate the expression of *Vrk2* in the thalamus, PCR was performed using Taq polymerase (Quest, USA) under the following conditions: 95°C for 3 min for the initial denaturation; 95°C for 30 s, 55°C for 30 s, 72°C for 30 s; in 32 cycles, followed by 72°C for 5 min for the final extension.

To analyze thalamic expression levels of *Vrk2*, tonic GABA‐related genes (*Gabard*, *Best1*, *Dao*, *Aldh1a1*, and *Maob*), and cell‐specific marker genes (*Aldh1l1*, *Rbfox3*, and *Aif1*). qPCR was performed using TB Green Premix Ex Taq II (Tli RNaseH Plus) (RR820A, Takara, Kusatsu, Japan). Conditions for qPCR were as follows: 95°C for 3 min for the initial denaturation, 95°C for 30 s, 55°C for 30 s, 72°C for 30 s; in 40 or 50 cycles, followed by 72°C for 5 min for the final extension. The recorded cycle (Ct) of *mouse β‐actin* was subtracted from the Ct of the other genes. Each relative Ct was normalized by its average. Based on PCR products that were duplicated by each PCR cycle, we measured the relative expression level by calculating the exponent of 2 to the power of the normalized Ct of each gene.

### 2.6 Immunohistochemistry

**FIGURE 2:**
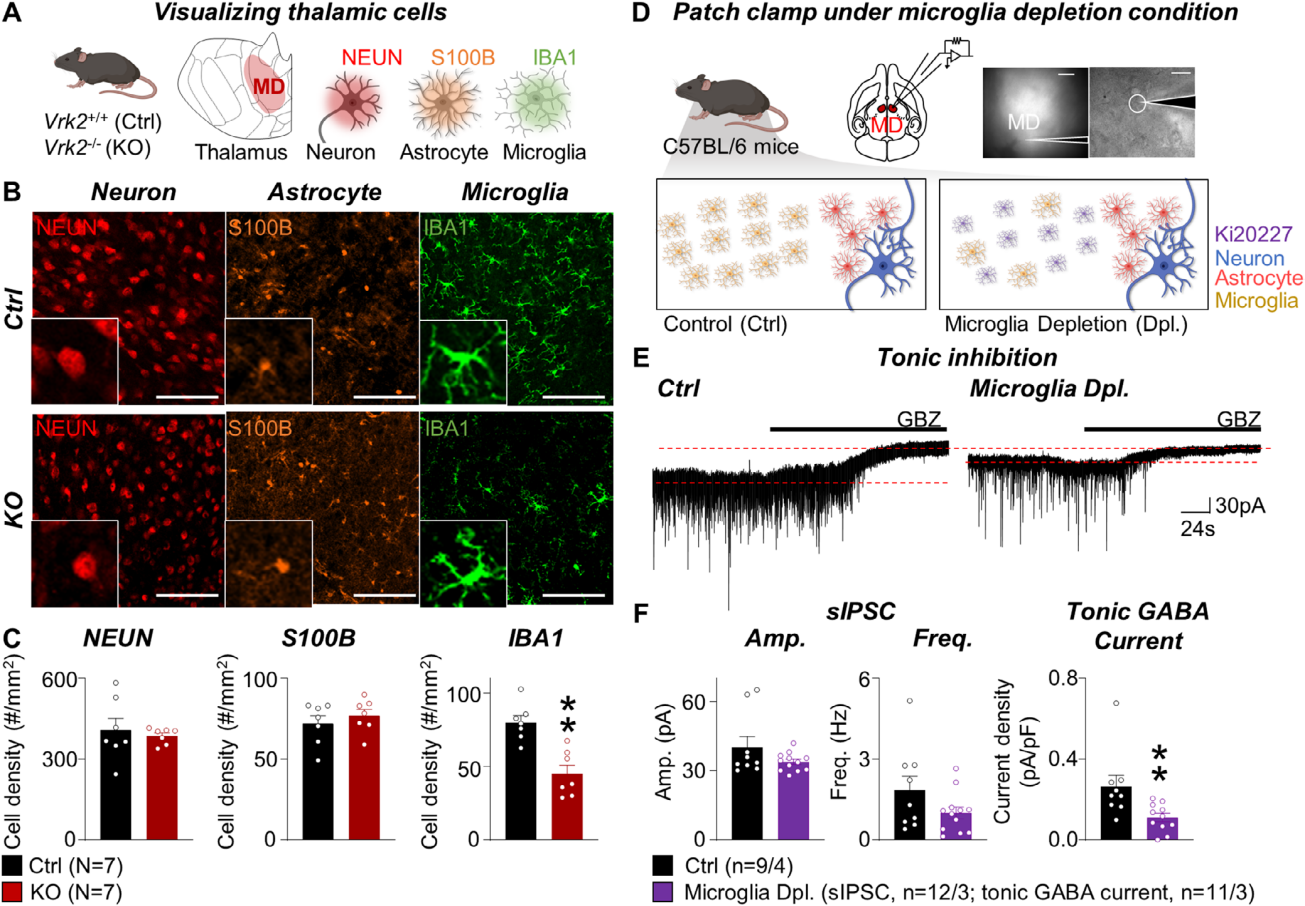
Loss of microglia in the *Vrk2*‐deficient mice reduces tonic GABA currents in the mediodorsal thalamus. (A) Schematic of visualizing neuron, astrocyte, and microglia in the control (*Vrk2* ^+/+^, Ctrl) and *Vrk2*‐deficient (*Vrk2* ^−/−^, KO) mice. (B) Representative images of NEUN (red) for neurons, S100B (orange) for astrocytes, and IBA1 (green) for microglia in mediodorsal (MD) thalamus of Ctrl and KO mice. Scale bar, 100 μm. (C) Quantification of cell density of NEUN^+^, S100B^+^, and IBA1^+^ cells. (D) Scheme and representative image for ex vivo electrophysiology in the MD thalamus (*top*). Scale bar, 200 and 20 μm. Schematic of wild‐type mice and microglia depletion (Dpl.) by Ki20227 administration (*bottom*). (E) Representative trace of tonic inhibition in control (Crtl) and microglia depletion (Microglia Dpl.) conditions. (F) Quantification of amplitude (Amp.), frequency (Freq.), and tonic GABA current in Ctrl and Microglia Dpl. conditions. ‘*N*’ denotes the number of mice (C). ‘*n*’ denotes the number of cells/mice (F). Data are presented as mean ± SEM (C and F). **p < 0.01, Mann–Whitney test (C and F). See also Figures S3 and S4.

Four to six weeks old male C57BL/6J, *Vrk2*‐deficient (for Figures 2 and S3), Ki20227‐administered (for Figure S4), and C87‐injected (for Figure S6) mice were anesthetized via i.p. injection of 0.2% tribromoethanol in saline in 20 mL/kg (A18706.14, Alfa Aesar, Ward Hill, MA, USA) and transcardially perfused with 1 M PBS, followed by a 4% paraformaldehyde (PFA; 158127, Sigma Aldrich, St. Louis, MO, USA) in PBS. The brains were isolated and post‐fixed in a 4% PFA solution overnight, followed by immersion in 30% sucrose in PBS for 72 h to achieve cryoprotection. The brains were mounted in O.C.T. compound (4583, Sakura Finetek, Torrance, CA, USA) and were sliced in a coronal section with a cryostat (CM1520, Leica, Wetzlar, Germany) to obtain brain sections (thickness: 40 μm) containing the thalamus.

The brain sections were washed three times with PBS and were permeabilized by a mixed solution containing 0.1% Tween 20 (P1379, Sigma Aldrich, St. Louis, MO, USA) and 5% normal goat serum (NGS; 005‐000‐121, Jackson ImmunoResearch, UK) in PBS for 30 min. Then, the brain sections were incubated for 1 h in 5% normal goat serum in PBS and overnight with primary antibodies at 4°C.

To visualize the cells in the *Vrk2*‐deficient mice, the indicated primary antibodies were used: mouse host anti‐NEUN (*RRID*: AB_2298772, MAB377, Sigma Aldrich, St. Louis, MO, USA), rabbit host anti‐S100B (*RRID*: AB_956280, ab41548, Abcam, Cambridge, UK), and rabbit host anti‐IBA1 (*RRID*: AB_839504, 019‐19741, Wako, Osaka, Japan). The brain sections were washed three times with PBS and incubated with the appropriate Alexa Fluor 488 AffiniPure Goat Anti‐Rabbit IgG (H + L) (*RRID: AB_2338046*, 111‐545‐003, Jackson ImmunoResearch, West Grove, PA, USA) for S100B and IBA1 and Cy3 AffiniPure Goat Anti‐Mouse IgG (H + L) (*RRID*: AB_2338680, 115‐165‐003, West Grove, PA, USA) for NEUN for 2 h at room temperature. After three washes with PBS, the brain sections were stained with DAPI and mounted onto glass slides (22‐037‐246, Thermo Fisher Scientific, Waltham, MA, USA) with mounting medium (S3023, Dako, Glostrup, Denmark). Images were taken at three random regions in the MD thalamus in each mouse using confocal microscopy (Carl Zeiss, Oberkochen, Germany; or Olympus, Tokyo, Japan). For the analysis of images, the cell number was analyzed, and the average number of counted cells from four images in each mouse was calculated.

To visualize cells in the Ki20227‐administered and C87‐injected mice, the primary antibodies were guinea pig host anti‐NEUN (*RRID*: AB_2298772, ABN90, Sigma Aldrich, St. Louis, MO, USA), rabbit host anti‐IBA1 (*RRID*: AB_839504, 019‐19741, Wako, USA), mouse host anti‐S100B (*RRID*: AB_956280, MA1‐25005, Invitrogen, USA), and chicken host anti‐GFAP (*RRID*: AB_177521, AB5541, Sigma Aldrich, St. Louis, MO, USA). The brain sections were washed three times with PBS and incubated with the appropriate Alexa Fluor 647 AffiniPure Goat Anti‐Mouse IgG (H + L) (*RRID*: AB_2338902, 115‐605‐003, Jackson ImmunoResearch, West Grove, PA, USA) for S100B, Alexa Fluor 488 AffiniPure Goat Anti‐Chicken IgY (IgG) (H + L) (*RRID*: AB_2337390, 103‐545‐155, Jackson ImmunoResearch, West Grove, PA, USA) for GFAP, Alexa Fluor 488 AffiniPure Goat Anti‐Rabbit IgG (H + L) (RRID: AB_2338046, 111‐545‐003, Jackson ImmunoResearch, West Grove, PA, USA) for IBA1, and Alexa Fluor 647 AffiniPure Goat Anti‐Guinea Pig IgG (H + L) (RRID: AB_2337446, 106‐605‐003, Jackson ImmunoResearch, West Grove, PA, USA) for NEUN for 2 h at room temperature. Subsequently, brain sections were washed, stained with DAPI, and analyzed as described previously. Images were acquired from the hippocampal CA1 and MD thalamus in each mouse using confocal microscopy (Carl Zeiss, Oberkochen, Germany; or Olympus, Tokyo, Japan).

To visualize astrocytes in the *Vrk2*‐deficient mice, the primary antibodies were mouse host anti‐S100B (*RRID*: AB_956280, MA1‐25005, Invitrogen, USA) and chicken host anti‐GFAP (*RRID*: *AB_177521*, AB5541, Sigma Aldrich, St. Louis, MO, USA). The brain sections were washed three times with PBS and incubated with the appropriate Alexa Fluor 488 AffiniPure Goat Anti‐Mouse IgG (H + L) (*RRID*: AB_2338840, 115‐545‐003, Jackson ImmunoResearch, West Grove, PA, USA) for S100B and Alexa Fluor 647 AffiniPure Goat Anti‐Chicken IgY (IgG) (H + L) (*RRID*: *AB_2337392*, 103‐605‐155, Jackson ImmunoResearch, West Grove, PA, USA) for GFAP for 2 h at room temperature. Subsequently, brain sections were washed and stained with DAPI as described previously. Images were acquired from the hippocampal CA1 and MD thalamus in each mouse using confocal microscopy (Carl Zeiss, Oberkochen, Germany; or Olympus, Tokyo, Japan).

### 2.7 Ex Vivo Electrophysiology

**FIGURE 3:**
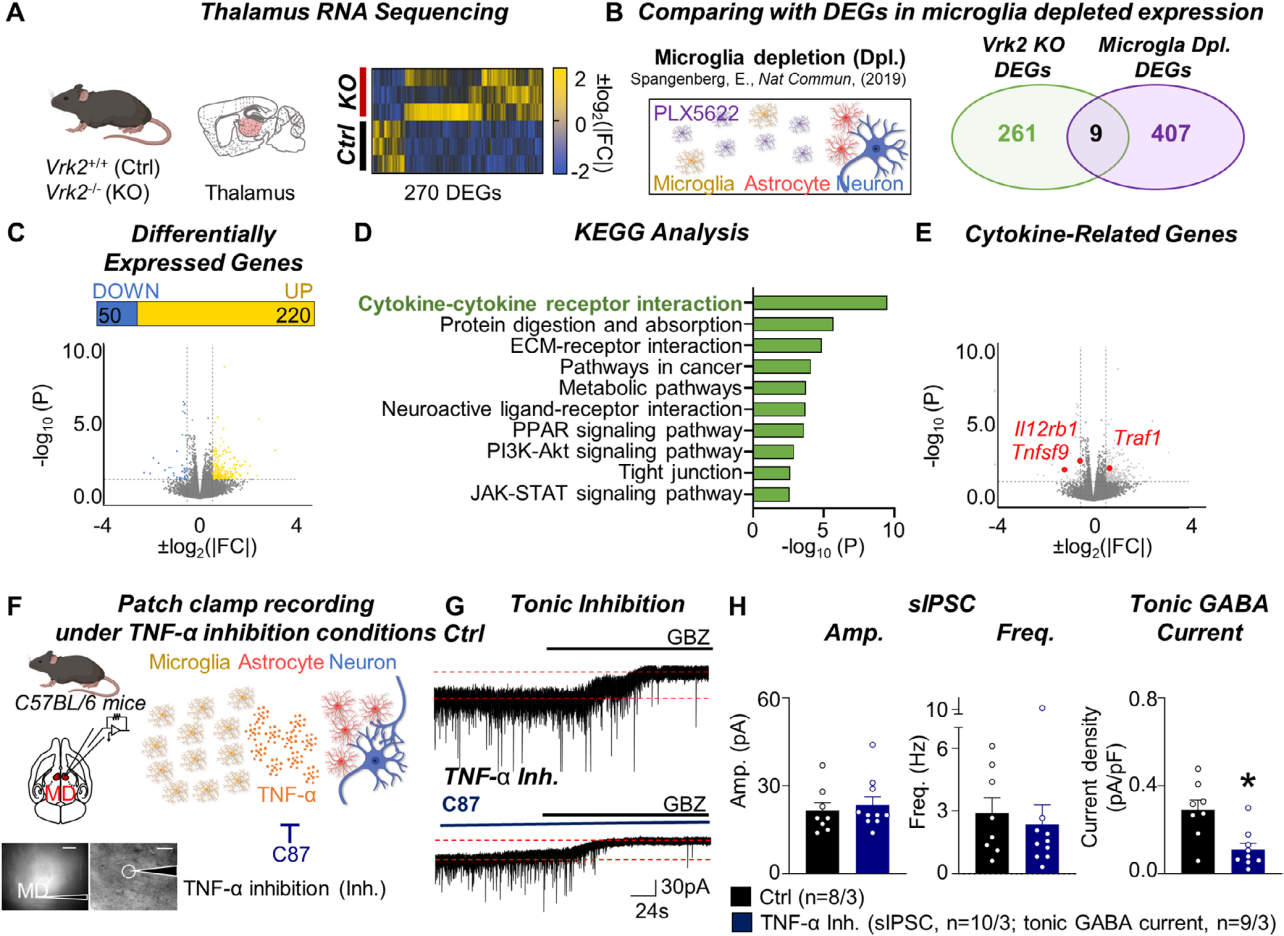
Disrupted cytokine pathways in the *Vrk2*‐deficient mice impair tonic GABA currents in the mediodorsal thalamus. (A) Schematic (*left*) and heat map (*right*) of 270 differentially expressed genes(DEGs) of the thalamus in the control (*Vrk2* ^+/+^, Ctrl) and *Vrk2*‐deficient (*Vrk2* ^−/−^, KO) mice. (B) Scheme (*left*) and Vandiagram (*right*) for cross‐referring DEGs in the KO mice and PLX5622‐treated mice [microglia depletion (Dpl.) group]. (C) The proportion (*top*) and volcano plot (*bottom*) for the upregulated (220 genes, yellow) and downregulated (50 genes, blue) DEGs, which are defined as fold change (FC) > 1.5 and *p*‐value (*P*) < 0.05, of bulk RNA sequencing in the Ctrl and KO mice. (D) Quantification of −log_10_(*P*) of gene expression in the top 10 KEGG pathways. (E) Volcano plot of ±log_10_(|FC|) and −log_10_(*P*) for *Tnfsf9*, *Il12rb1*, and *Traf1*. (F) Scheme and representative image for ex vivo electrophysiology in the mediodorsal (MD) thalamus (*left*). Scale bar, 200 and 20 μm. Schematic (*right*) of TNF‐α inhibition (Inh.) by C87 application. (G) Representative trace of tonic inhibition in wild‐type (Ctrl) and TNF‐α inhibition (TNF‐α Inh.) condition. (H) Quantification of amplitude (Amp.), frequency (Freq.), and tonic GABA current in Ctrl and TNF‐α Inh. conditions. ‘*n*’ denotes the number of cells/mice (H). Data are presented as mean ± SEM (H). *p < 0.05, Mann–Whitney test (H). See also Figures S5 and S6.

**FIGURE 4:**
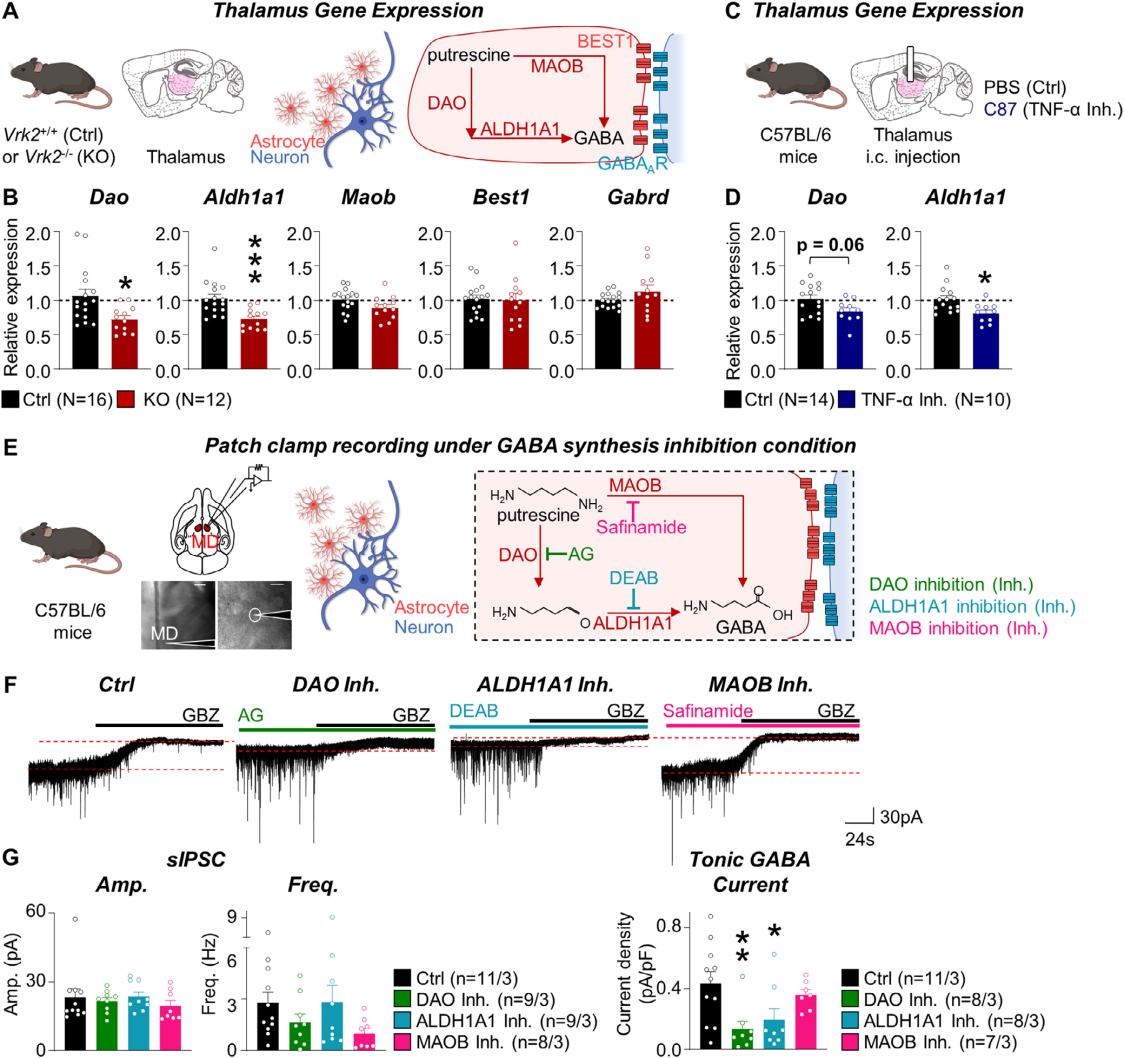
The DAO–ALDH1A1 pathway is disrupted in *Vrk2*‐deficient thalamus, reducing tonic GABA currents in the mediodorsal thalamus. (A) Schematic of quantitative PCR (qPCR) analysis of the thalamus in the control (*Vrk2* ^+/+^, Ctrl) and *Vrk2*‐deficient (*Vrk2* ^−/−^, KO) mice (*left*) and astrocyte‐derived GABA‐related genes (*right*); diamine oxidase (DAO), aldehyde dehydrogenase 1 family member a1 (ALDH1A1), monoamine oxidase b (MAOB), bestrophin 1 (BEST1), and GABA a receptor (GABA_A_R). (B) Quantification of gene expression of *Dao*, *Aldh1a1*, *Maob*, *Best1*, and *Gabrd*. (C) Schematic diagrams for qPCR analysis of the thalamus in the saline‐injected control (Ctrl) and C87‐injected TNF‐α inhibition (Inh.) mice. (D) Quantification of gene expression of *Dao* and *Aldh1a1*. (E) Scheme and representative image for ex vivo electrophysiology in the mediodorsal (MD) thalamus (*left*). Scale bar, 200 and 20 μm. Schematic for GABA synthesis enzyme inhibition (Inh.) by aminoguanidine (AG) for DAO, diethylaminobenzaldehyde (DEAB) for ALDH1A1, and safinamide for MAOB application on wild‐type mice (*right*). (F) Representative trace of tonic inhibition of wild‐type mice (Ctrl) with DAO Inh., ALDH1A1 Inh., and MAOB Inh. conditions. (G) Quantification of tonic inhibition current in Ctrl, DAO Inh., ALDH1A1 Inh., and MAOB Inh. conditions. ‘*N*’ denotes the number of mice (B and D). ‘*n*’ denotes the number of cells/mice (E). Data are presented as mean ± SEM (B, D, and G). *p < 0.05, **p < 0.01, and ***p < 0.05; Mann–Whitney test (B and D) and one‐way ANOVA test (G).

The 4–6 week‐old male C57BL/6J (for Figures 3 and 4), *Vrk2*‐deficient (for Figures 1 and S2), and Ki20227 administered (for Figure 2) mice were anesthetized with 2‐bromo‐2‐chloro‐1,1,1‐trifluoroethane by inhalation. The mouse brain was prepped in the ice‐cold sectioning solution containing (in mM): 234 sucrose, 2.5 KCl, 10 MgSO_4_, 1.25 NaH_2_PO_4_, 24 NaHCO_3_, 0.5 CaCl_2_–H_2_O, and 11 Glucose with 95% O_2_ and 5% CO_2_ (310–320 mOsm). The 300 μm horizontal slice containing the thalamus was cut in ice‐cold sectioning solution on a vibrating microtome (Leica, Wetzlar, Germany). The collected slices were incubated for an hour in incubating solution containing (in mM): 124 NaCl, 3 KCl, 6.5 MgSO_4_, 1.25 NaH_2_PO_4_, 26 NaHCO_3_, 10 D‐ (^+^)‐glucose, 1 CaCl_2_, saturated with 95% O_2_ and 5% CO_2_ (310–320 mOsm). To record tonic GABA current in the inhibition of tonic GABA‐related molecules, the collected slices were incubated for at least 2 h in incubating solution with 10 μM aminoguanidine (AG; 396494, Sigma‐Aldrich, St. Louis, MO, USA), 10 μM diethylaminobenzaldehyde (DEAB; D86256, Sigma‐Aldrich, St. Louis, MO, USA), or 1 μM safinamide (provided by the Korea Institute of Science and Technology, KIST) (Kwak et al. 2020; Park et al. 2019).

The slices were transferred to a recording chamber and continuously perfused with artificial cerebrospinal fluid (aCSF) containing (in mM): 124 NaCl, 3 KCl, 1.3 MgSO_4_, 1.25 NaH_2_PO_4_, 26 NaHCO_3_, 10 D‐(+)‐glucose, 2.4 CaCl_2_, saturated with 95% O_2_ and 5% CO_2_ (310–320 mOsm). Neurons were visualized with infrared light on an upright microscope (Olympus, Tokyo, Japan) with HC Image (RRID: SCR_015041, Hamamatsu Photonics, Shizuoka, Japan). Recording glass pipettes from a pipette puller (P‐97, Sutter Instrument, Novato, CA, USA) with 2–6 MΩ resistance were used for recording. To record tonic GABA current and inhibitory postsynaptic currents (IPSCs) in the thalamic neurons, pipettes were filled with CsCl intracellular solution containing (in mM): 135 CsCl, 10 HEPES, 10 EGTA, 5 Mg^2+^‐ATP, 0.5 Na^+^‐GTP with 5 mM QX‐314 (L5783, Sigma‐Aldrich, St. Louis, MO, USA) (280–290 mOsm, pH 7.3–7.4). Before recording, the baseline current was stabilized with 50 μM APV (0106, Tocris Bioscience, Bristol, UK) and 50 μM CNQX (1045, Tocris Bioscience, Bristol, UK) application in aCSF, and the spontaneous IPSCs were recorded over 5 min at −60 mV. After recording IPSCs, the amplitude of the tonic GABA current was measured by the baseline shift after 10 μM gabazine (S106, Sigma Aldrich, St. Louis, MO, USA) application in aCSF. To record tonic GABA current and IPSCs in the TNF‐α inhibition condition, C87 (40 μM) was applied in every procedure (Yamamoto et al. 2019). To record excitatory postsynaptic currents (EPSCs) in the thalamus, pipettes were filled with K^+^‐gluconate intracellular solution containing (in mM): 145 K^+^‐gluconate, 10 HEPES, 5 NaCl, 0.2 EGTA, 5 Mg^2+^‐ATP, 0.5 Na^+^‐GTP with 5 mM QX‐314 (280–290 mOsm, pH 7.3–7.4). Before recording, the baseline current was stabilized with 10 μM gabazine application in aCSF, and the spontaneous EPSCs were recorded over 5 min at −60 mV. To record intrinsic excitability in the thalamus, pipettes were filled with K‐gluconate intracellular solution. The resting membrane potential (RMP) was recorded without current injection. Then, manipulate the baseline potential to −58 mV to distinguish the burst firing from tonic firing without being mixed. To record tonic firing, action potentials were generated by injecting 1‐s current steps from 0 to 200 pA (increasing by 20 pA) in the MD thalamus. To record bursting, a 1‐s current pulse that induces hyperpolarization to about −80 mV was used to elicit an action potential.

Signals were amplified using a MultiClamp amplifier (700B, Molecular Devices, San Jose, CA, USA) and MultiClamp software (RRID: SCR_018455, Molecular Devices, San Jose, CA, USA), and data acquisition was performed using a digitizer (1550B, Molecular Devices, USA) and pClamp (RRID: SCR_011323, Molecular Devices, San Jose, CA, USA). Cells that had a series resistance exceeding 30 MΩ were excluded. The action potentials in the tonic and burst firing were detected in standards of peak amplitudes (> 0 mV) and initial slope (> 20 mV/ms), and spike numbers were measured as the total number of spikes in each step. The frequency and amplitude of spontaneous IPSCs (sIPSCs) and EPSCs (sEPSCs) were analyzed using Minianalysis (RRID: SCR_002184, Synaptosoft, Fort Lee, NJ, USA).

### 2.8 Data Analysis

The number of experimental samples, mean, and SEM values is described in the figure legend. For data presentation and statistical analysis, GraphPad Prism (RRID: SCR_002798, GraphPad Software, San Diego, CA, USA) was used. For electrophysiology, Minianalysis and pClamp were used. For image analysis, ImageJ software (RRID: SCR_003070) with the Cell Counter plugin was used. Statistical significance was set at *p < 0.05, **p < 0.01, ***p < 0.001 and ****p < 0.0001. Data are presented as mean ± SEM and not detected (n.d.).

## 3 Results

### 3.1 VRK2 Contributes to Synaptic and Extra‐Synaptic Transmission in the MD Thalamus

To investigate the role of VRK2 in thalamic circuit function, we first assessed its expression and genetic deletion in the thalamus. Quantitative PCR in the thalamus tissues displayed robust *Vrk2* expression in wild‐type mice and complete loss in *Vrk2*‐deficient mice (Ctrl, 1.02 ± 0.05, *N* = 16; KO, 0.14 ± 0.05; *N* = 13; ****p < 0.0001) (Figure 1A,B), validating the knockout model for functional studies in this region.

To determine the cellular identity of *Vrk2*‐expressing cells in the thalamus, we performed single‐cell RNA sequencing (scRNA) on acutely dissected thalamic tissues and transcriptomic profiling to identify *Vrk2*‐expressing cell populations (Figure 1C). Using well‐established markers, we classified major cell populations: neurons by *Rbfox3*, *Snap25*, *Syt1*, and *Gria2*; astrocytes by *Gja1*, and *Aldh1l1*; microglia by *C1qa*, *P2ry12*, and *Trem2*; oligodendrocytes by *Mbp* and *Plp1*; endothelial cells by *Flt1*, *Cldn5*, *Kdr*, and *Pecam1*; epithelial cells by Cdh1, Ocln, Cldn family, and krt family; perivascular cells by *Kcnj8* and *Abcc9*; vascular smooth muscle by *Acta2* and *Tagln* (Figure 1C). *Vrk2* expression was highly enriched in microglia, with negligible expression in neurons or astrocytes (Neuron, 0.03 ± 0.008, *n* = 659/3; Astrocyte, 0.03 ± 0.004, *n* = 3034/3; Microglia, 0.20 ± 0.013, *n* = 1831/3; Oligodendrocyte, 0.03 ± 0.006, *n* = 840/3; Endothelial cell, 0.04 ± 0.008, *n* = 881/3; Epithelial cell, 0.05 ± 0.009, *n* = 324/3; Perivascular cell, 0.04 ± 0.021, *n* = 104/3; Vascular smooth muscle, 0.06 ± 0.022, *n* = 199/3) (Figure 1D). Consistent with these transcriptomic data, fluorescence‐activated cell sorting (FACS) followed by quantitative PCR confirmed that CD11b^+^ microglia isolated from the thalamus showed markedly higher *Vrk2* expression compared to other cell populations (Figure S1A–C). Notably, microglial *Vrk2* levels increased with age, implying a role for VRK2 in both developmental and post‐developmental microglial functions (Figure S1D). This cell‐type specificity suggests that VRK2 may regulate thalamic function primarily through microglia‐associated mechanisms.

To assess whether VRK2 influences the physiological properties of thalamocortical neurons in the MD thalamus, we measured the resting membrane potential and action potential firing in response to stepwise depolarizing and hyperpolarizing current injections, including tonic firing and burst firing (Figure 1E,F). All parameters were comparable between *Vrk2*‐deficient and wild‐type mice (Figure 1G–J), indicating that *Vrk2* deletion does not affect the basic excitability of MD thalamic neurons.

Given the dense synaptic connectivity of MD thalamic neurons, we next asked whether synaptic transmission onto these neurons is affected in the absence of VRK2. Recordings of sEPSCs in MD neurons revealed a significant reduction in frequency (Ctrl, 0.72 ± 0.15 Hz, *n* = 9/3; KO, 0.24 ± 0.06 Hz, *n* = 7/3; **p = 0.0079), while the amplitude remained unchanged (Figure 1K–M). These findings align with previous work showing that VRK2 deficiency reduces the frequency of miniature EPSC (mEPSC) and spontaneous EPSC (sEPSC), indicating a decrease of functional excitatory synapses by microglial pruning in the hippocampus (Lee et al. 2019). In contrast, sIPSCs were unchanged in both frequency and amplitude (Figure 1N–P), indicating that inhibitory input is preserved. This selective impairment of excitatory transmission is consistent with studies showing that microglia preferentially prune excitatory synapses (Lee et al. 2021).

In addition to phasic synaptic inputs, thalamic neurons are regulated by tonic inhibition—an extrasynaptic process driven by astrocytic GABA release. This tonic inhibition plays a critical role in modulating thalamic excitability and circuit function (Ju et al. 2022; Koh et al. 2023; Kwak et al. 2020; Paydar et al. 2014). To assess whether VRK2 affects this mechanism, we measured tonic GABA currents in MD thalamic neurons by quantifying the baseline shift in holding current after GABA_A_ receptor blockade (Figure 1Q–R). Surprisingly, tonic GABA current was significantly reduced in the *Vrk2*‐deficient mice (Ctrl, 0.28 ± 0.04 pA/pF, *n* = 15/6; KO, 0.15 ± 0.05 pA/pF, *n* = 8/4; *p = 0.0385) (Figure 1S), indicating a reduction in extracellular GABA tone. To test whether this effect extended beyond the MD thalamus, we also examined tonic inhibition in the ventrobasal (VB) thalamus—a first‐order sensory relay nucleus. Similar reductions were observed in VB thalamocortical neurons (Figure S2), suggesting that VRK2 contributes broadly to tonic inhibition across thalamic subregions.

Together, these results demonstrate that VRK2 plays a dual role in MD thalamic circuit regulation. First, VRK2 is required for maintaining excitatory synaptic input onto MD thalamocortical neurons, likely through microglia‐mediated synaptic pruning. Second, VRK2 is essential for sustaining astrocyte‐mediated tonic GABA inhibition, despite being expressed exclusively in microglia. These results point to a novel trans‐cellular mechanism in which microglial VRK2 modulates astrocytic GABA output, revealing a glial–glial axis critical for maintaining thalamic circuit function and potentially underlying cognitive impairments in VRK2‐associated neurodevelopmental disorders.

### 3.2 Microglial Loss Impairs Tonic Inhibition in the MD Thalamus

Building on our finding that VRK2 deficiency disrupts astrocyte‐mediated tonic inhibition in the MD thalamus, we next asked whether this effect could be attributed to altered microglial function. Given the microglia‐specific expression of *Vrk2* (Lee et al. 2019), we hypothesized that VRK2 regulates tonic inhibition indirectly, through microglia‐dependent signaling mechanisms.

To assess this possibility, we first examined whether *Vrk2* deficiency alters cellular composition within the MD thalamus. We performed immunostaining using NEUN for neurons, S100B for astrocytes, and IBA1 for microglia to quantify cell populations. While the numbers of NEUN^+^ cells and S100B^+^ cells remained unchanged, IBA1^+^ cell density was significantly reduced in *Vrk2*‐deficient mice, suggesting microglial density is reduced by *Vrk2* deficiency (Ctrl, 79.88 ± 4.77 #/mm^2^, *N* = 7; KO, 45.05 ± 5.85 #/mm^2^, *N* = 7; **p = 0.0012) (Figure 2A–C). Notably, the remaining microglia retained a ramified morphology indicative of a homeostatic, non‐reactive state, suggesting that the reduction in microglial number was not due to inflammatory activation (Green and Rowe 2024). Astrocytes also displayed a non‐reactive phenotype in *Vrk2*‐deficient mice. In the thalamus, where astrocytes typically show minimal or no GFAP expression under physiological conditions, GFAP immunoreactivity remained absent despite normal astrocyte density in *Vrk2*‐deficient mice (Figure S3A–C). In contrast, hippocampal astrocytes—which constitutively express GFAP under baseline conditions—exhibited normal morphology and unchanged cell number in *Vrk2*‐deficient mice (Figure S3D,E). These findings confirm that astrocytes in both the thalamus and hippocampus remain in a homeostatic, non‐reactive state. Together, these results suggest that *Vrk2* is required for maintaining the microglial population in the MD thalamus under non‐inflammatory conditions.

To directly test whether microglia are functionally required for tonic GABA regulation, we selectively depleted microglia in wild‐type mice using oral administration of the CSF1R inhibitor, Ki20227, with water for 7 consecutive days, following established protocols (Yamamoto et al. 2019) (Figure 2D). Ki20227 treatment efficiently ablated by approximately 97% in the MD thalamus (Figure S4A–C) and by ~90% in the hippocampal CA1 (Figure S4D,E). Microglia depletion resulted in a significant reduction in tonic GABA currents in MD thalamic neurons (Ctrl, 0.26 ± 0.06 pA/pF, *n* = 9/4; Microglia Dpl., 0.11 ± 0.02 pA/pF, *n* = 11/3; **p = 0.0023) (Figure 2E,F). In contrast, phasic inhibition—as assessed by sIPSC amplitude and frequency—remained unaltered, indicating that microglia specifically support extra‐synaptic GABA tone without affecting synaptic GABA transmission. Thus, microglia are essential for sustaining tonic GABAergic inhibition in the MD thalamus.

Taken together, these findings demonstrate that microglia are functionally required to maintain astrocyte‐mediated tonic inhibition in the MD thalamus. Combined with the microglia‐specific expression of *Vrk2*, these data support a model in which VRK2 indirectly regulates tonic GABA tone by maintaining microglial integrity and function, which in turn influences astrocytic GABA output.

### 3.3 VRK2 Regulates Microglial Cytokine Signaling to Support Tonic Inhibition

To investigate how VRK2 shapes microglial function at the molecular level, we performed bulk RNA‐sequencing of thalamic tissue from *Vrk2*‐deficient and wild‐type mice (Figure 3A). Differential expression analysis revealed 270 significantly altered genes (|fold change| > 1.5 and *p*‐value < 0.05) (Figure 3A). Because *Vrk2*‐deficient mice exhibit reduced microglial density, we compared our transcriptomic results with a published RNA sequencing dataset from wild‐type mice treated with the CSF1R inhibitor PLX5622, which induces microglial depletion in the thalamus and striatum (Spangenberg et al. 2019) (https://rnaseq.mind.uci.edu/green/ad_plx/). Cross‐referencing the differentially expressed genes (DEGs) between the two datasets revealed that only nine genes (*Nlrp3*, *Hk3*, *Nrurl3*, *Ms4a6c*, *Sec14l3*, *Ermap*, *Erdr1*, *Itgb7*, and *Slc6a3*) overlapped (Figure 3B). This minimal overlap indicates that the vast majority of transcriptional changes in *Vrk2*‐deficient mice arise from VRK2 loss itself rather than secondary effects of microglial depletion. Moreover, the direction of change for these nine shared genes was not consistent between the *Vrk2*‐deficient and PLX5622‐treated groups, further confirming that the two conditions represent fundamentally different molecular states. Among the DEGs in *Vrk2*‐deficient mice, 220 genes were upregulated and 50 were downregulated (Figure 3C). KEGG enrichment analysis identified that the most significantly affected pathway was cytokine–cytokine receptor interaction [−log_10_(*P*) = 9.50], followed by pathways related to protein digestion, ECM–receptor interaction, neuroactive ligand signaling, and JAK–STAT and PI3K‐Akt signaling (Figures 3D and S5). These findings suggest that cytokine dysregulation is a major molecular consequence of VRK2 loss in the thalamus.

Among the DEGs, several cytokine signaling components, including *Tnfsf9* and *Il12rb1*, were downregulated (Figure 3E), despite the fact that baseline expression of cytokines in non‐reactive microglia is minimal under homeostatic conditions and typically increases during inflammation (Li and Barres 2018; Shastri et al. 2013). The previous studies show that *Tnfsf9*, expressing 4‐1BBL, one of the tumor necrosis factor (TNF) superfamily, binds to 4‐1BB in immune cells, amplifying cytokine release via TRAF‐mediated NF‐kB pathways (Watts et al. 2025). Similarly, *Il12rb1* is essential for IL‐12 signaling through the JAK2–STAT4 pathway, a major route promoting IFN‐γ production and M1‐like activation in CNS‐resident immune cells (Tait Wojno et al. 2019). In contrast, *Traf1*, a negative regulator of the TNF signaling pathway, was upregulated in *Vrk‐2*‐deficient mice (Figure 3E), consistent with suppression of canonical inflammatory signaling (Tsitsikov et al. 2001). These results align with previous findings that VRK2, as a serine/threonine kinase, phosphorylates NFAT1 to promote COX‐2 expression and thereby upregulate cytokines such as interleukins and TNF‐α (Canellada et al. 2006; Lee et al. 2018; Vazquez‐Cedeira and Lazo 2012). Collectively, these data suggest that VRK2 orchestrates the molecular output of thalamic microglia by tuning cytokine‐related signaling, particularly within the TNF superfamily. Such signaling may be required for maintaining astrocyte–neuronal communication and extracellular inhibitory tone.

Given that TNF‐α is a well‐established mediator of microglia–astrocyte crosstalk (Jha et al. 2019; Matejuk and Ransohoff 2020), we tested whether inhibiting TNF‐α signaling would directly impact tonic GABA levels. To test this, we applied C87, a selective TNF‐α inhibitor (Yamamoto et al. 2019), to acute brain slices and incubated them for 2 h before electrophysiological recordings (Figure 3F). Tonic GABA currents in MD thalamic neurons were significantly reduced following C87 treatment (Ctrl, 0.29 ± 0.05 pA/pF, *n* = 8/3; TNF‐α Dpl.; 0.11 ± 0.03 pA/pF, *n* = 9/3 *p = 0.0111) (Figure 3G,H), closely phenocopying the reductions observed in both *Vrk2*‐deficient and microglia‐depleted mice. Importantly, microglial density in the thalamus remained unchanged after C87 treatment (Figure S6), confirming that the reduction in tonic GABA was not secondary to microglial loss. These findings demonstrate that TNF‐α signaling is indispensable for sustaining tonic GABA levels, identifying it as a key molecular link in microglia–astrocyte communication.

Together, these results demonstrate that VRK2 supports tonic inhibition in the MD thalamus by regulating microglial cytokine signaling, particularly through the TNF‐α pathway. Disruption of this signaling cascade impairs astrocytic GABA synthesis or release, leading to weakened tonic inhibition. These findings highlight a previously unrecognized mechanism in which VRK2 orchestrates the microglia–astrocyte–neuron axis essential for maintaining thalamic inhibitory tone.

### 3.4 Astrocytic GABA Synthesis is Impaired in *Vrk2*‐Deficient Mice and Underlies Reduced Tonic Inhibition in the MD Thalamus

Given the critical role of microglia–astrocyte signaling in modulating tonic inhibition, and the disruption of microglial cytokine output observed in *Vrk2*‐deficient mice, we next investigated whether astrocyte‐derived GABA synthesis is itself impaired in the MD thalamus. Astrocytes generate GABA from putrescine via two main enzymatic routes: (1) the diamine oxidase (DAO) and aldehyde dehydrogenase 1a1 (ALDH1A1) pathway, and (2) the monoamine oxidase B (MAOB) pathway (Figure 4A) (Koh et al. 2023; Kwak et al. 2020). Once synthesized, GABA is released through the GABA‐permeable bestrophin1 (BEST1) channel, where it acts on extrasynaptic GABA_A_ receptors—particularly those containing the δ subunit (GABARD)—to generate a persistent inhibitory current known as tonic inhibition (Figure 4A) (Koh et al. 2023; Kwak et al. 2020). This form of inhibition modulates neuronal excitability by reducing membrane resistance and time constants, without requiring membrane hyperpolarization (Kwak et al. 2020).

To determine whether these molecular pathways are affected by *Vrk2* deletion, we performed quantitative PCR on thalamic tissue to assess the expression of genes involved in astrocytic GABA synthesis (*Dao*, *Aldh1a1*, and *Maob*), GABA release (*Best1*), and extrasynaptic receptor composition (*Gabrd*). Expression of both *Dao* and *Aldh1a1* was significantly reduced in *Vrk2*‐deficient mice (*Dao*; Ctrl, 1.06 ± 0.10, *N* = 16; KO; 0.72 ± 0.05, *N* = 12; *p = 0.013; *Aldh1a1*; Ctrl, 1.03 ± 0.06, *N* = 16; KO; 0.73 ± 0.04, *N* = 12; ***p = 0.0007), whereas *Maob*, *Best1*, and *Gabrd* levels remained unchanged (Figure 4B). Furthermore, thalamic *Dao* and *Aldh1a1* expression were similarly reduced following C87‐mediated TNF‐α inhibition (Figure 4C,D), establishing a causal link between microglial cytokine signaling and astrocytic GABA synthesis. These results suggest that *Vrk2* deletion selectively impairs the DAO–ALDH1A1 pathway responsible for GABA production without altering release machinery or receptor expression.

To functionally validate these molecular findings, we performed pharmacological manipulations during whole‐cell patch‐clamp recordings in the MD thalamus (Figure 4E,F). Acute slices were incubated for 2 h with AG to inhibit DAO, DEAB to inhibit ALDH1A1, or safinamide to inhibit MAOB (Kwak et al. 2020; Yamamoto et al. 2019). Inhibition of DAO or ALDH1A1 led to a substantial reduction in tonic GABA current, consistent with the observed transcriptional downregulation in *Vrk2*‐deficient tissue (Ctrl, 0.43 ± 0.08 pA/pF, *n* = 11/3; DAO inhibition; 0.13 ± 0.05 pA/pF, *n* = 8/3; **p = 0.0090; ALDH1A1 inhibition; 0.20 ± 0.07 pA/pF, *n* = 8/3; *p = 0.0314) (Figure 4G). In contrast, inhibition of MAOB had no significant effect on tonic current amplitude. These findings demonstrate that the reduced tonic inhibition in *Vrk2*‐deficient mice arises primarily from impaired astrocytic GABA synthesis through the DAO–ALDH1A1 pathway.

Together, they reveal a glia–glia signaling axis in which microglial VRK2‐dependent cytokine signaling regulates astrocytic metabolism to sustain tonic inhibition in the MD thalamus—a mechanism potentially underlying thalamocortical dysfunction in VRK2‐associated neurodevelopmental disorders.

## 4 Discussion

In this study, we uncovered a novel glial mechanism by which the NDD risk gene *Vrk2* regulates thalamic circuit function, particularly within the MD thalamus. Using *Vrk2*‐deficient mice, we demonstrate that VRK2 is essential for maintaining tonic inhibition and excitatory synaptic transmission in the MD thalamus (Figure 1). Our findings further reveal that this regulation occurs via a glial signaling axis: VRK2, which is selectively expressed in microglia, modulates astrocytic GABA synthesis through TNF‐α‐dependent cytokine signaling (Figures 2, 3, 4).

The synaptic and glial deficits observed in both the MD and VB thalamus suggest a broad role for VRK2 in maintaining thalamic excitability and balance (Figures 1 and S2). These alterations align with the wide range of cognitive and sensory functions often disrupted in NDDs (Lee et al. 2019). The mutation of *Vrk2* is considered a risk variant for schizophrenia (rs2312147, rs3732136) and epilepsy (rs1302641) patients (Consortium et al. 2012; Li, Wang, et al. 2012; Steinberg et al. 2011; Zhang et al. 2015). Correspondingly, *Vrk2*‐deficient mice exhibit phenotypes that recapitulate human NDD features, including deficits in social interaction, fear memory, and spatial learning (Lee et al. 2019). Dysfunction of thalamocortical connectivity has been repeatedly reported in youth and young adults at elevated clinical risk (Anticevic et al. 2015), as well as in individuals with ASD (Nair et al. 2013), and schizophrenia (Chen et al. 2019; Giraldo‐Chica et al. 2018; Pergola et al. 2015; Woodward et al. 2012). The MD thalamus specifically is critical for cognitive processes such as social behavior (Ferguson and Gao 2018; Karimi et al. 2021), social dominance (Zhou et al. 2017), cognitive flexibility (Anastasiades et al. 2021; Collins et al. 2018; Ferguson and Gao 2018), fear extinction (Lee et al. 2011; Paydar et al. 2014), and cognitive function (Karimi et al. 2021)—domains that are impaired in *Vrk2*‐deficient mice and NDDs more broadly (Anticevic et al. 2014; Giraldo‐Chica et al. 2018; Lee et al. 2019). Thus, the observed reductions in synaptic excitation and tonic inhibition in the MD thalamus may directly contribute to the behavioral phenotypes associated with *Vrk2* mutation.

In the MD thalamus, we found that VRK2 contributes to synaptic balance without altering the intrinsic excitability of thalamocortical neurons (Figure 1). In particular, *Vrk2*‐deficient mice exhibited a marked reduction in excitatory synaptic input, whereas inhibitory phasic input remained unchanged. This is consistent with prior findings in the hippocampus, where microglial VRK2 regulates excitatory synaptic pruning (Lee et al. 2019), and with studies showing that microglia preferentially eliminate excitatory over inhibitory synapses (Lee et al. 2021). Given that excitatory inputs to the MD thalamus predominantly arise from deep‐layer cortical pyramidal neurons (Sherman 2007; Sherman and Guillery 2011). Especially, the reciprocal excitatory synaptic interactions between the prefrontal cortex and MD thalamus are essential for working memory, social preference, social novelty, and social hierarchy (Anastasiades et al. 2021; Collins et al. 2018; Ferguson and Gao 2018; Zhou et al. 2017). Our findings suggest that microglial VRK2 may shape cortical–thalamic connectivity via selective control of excitatory synapse refinement.

Beyond synaptic inputs, our findings reveal that VRK2 plays a critical role in sustaining astrocyte‐mediated tonic GABA release in both the MD and VB thalamus (Figures 1 and S2). In the thalamus, astrocytes are now recognized as the principal source of tonic GABA, which plays a critical role in modulating the excitability and sensory responsiveness of thalamocortical neurons (Koh et al. 2023; Kwak et al. 2020) and also contributes to cognitive regulation, such as fear extinction learning (Paydar et al. 2014). Astrocytes synthesize GABA via two major pathways: the DAO–ALDH1A1 pathway and the MAOB pathway, using putrescine as a substrate (Ju et al. 2022; Koh et al. 2023; Kwak et al. 2020). GABA is then released via the calcium‐dependent BEST1 channel and acts on extrasynaptic GABA_A_ receptors containing the δ subunit (GABRD), generating a persistent tonic inhibitory current (Ju et al. 2022; Koh et al. 2023; Kwak et al. 2020). Our data show that *Vrk2* deficiency reduces the expression of *Dao* and *Aldh1a1*—but not *Maob*, *Best1*, or *Gabrd*—in the thalamus (Figure 4), and pharmacological inhibition of DAO and ALDH1A1 recapitulates the reduction in tonic current, implicating this pathway in the VRK2‐dependent regulation of astrocytic GABA synthesis.

Since VRK2 is not expressed in astrocytes (Figure 1), our data implicate a transcellular mechanism involving microglial regulation of astrocytic function. We found that VRK2 loss leads to reduced microglial density in the MD thalamus (Figure 2) and significantly alters microglial gene expression, particularly in cytokine signaling pathways (Figure 3). Bulk RNA‐seq revealed downregulation of *Tnfsf9* and *Il12rb1*, and upregulation of *Traf1*, consistent with disruption of TNF superfamily signaling (Tait Wojno et al. 2019; Tsitsikov et al. 2001; Watts et al. 2025). These findings build on prior studies showing that VRK2 phosphorylates NFAT1 to induce cytokine production, including TNF‐α (Canellada et al. 2006; Lee et al. 2018; Vazquez‐Cedeira and Lazo 2012). Although *Vrk2* deletion also reduces microglial density, the minimal overlap between the transcriptomic changes in *Vrk2*‐deficient mice and those induced by pharmacological microglial depletion (only nine shared genes; Figure 3) indicates that the majority of differentially expressed genes are driven by VRK2 loss itself rather than cell depletion. Moreover, ex vivo inhibition of TNF‐α signaling recapitulated the tonic GABA deficit observed in *Vrk2*‐deficient mice (Figure 3), while leaving microglial density unaffected (Figure S6). These findings identify TNF‐α as the critical effector linking microglia VRK2 signaling to astrocytic GABA synthesis, demonstrating that cytokine signaling—rather than microglial abundance per se—underlies the loss of tonic inhibition.

Microglia–astrocyte interactions are increasingly recognized as essential for CNS homeostasis. While these interactions are best known in inflammatory contexts (Jha et al. 2019; Matejuk and Ransohoff 2020), our findings show that microglia regulate astrocytic GABA synthesis under non‐reactive, homeostatic conditions. Microglial TNF‐α signaling appears necessary to sustain astrocytic GABA output and, consequently, tonic inhibition in the thalamus. This insight expands the functional repertoire of microglial cytokines and reveals a novel mechanism for maintaining synaptic and extrasynaptic balance.

Although direct molecular links between TNF‐α and the DAO‐ALDH1A1 pathway remain to be elucidated, prior studies suggest that TNF‐α signaling through astrocytic TNFR1/2 activates downstream cascades such as caspases, JNK–AP1, MAPK, and NF‐kB through TRAF1 and TRAF2 (Tsitsikov et al. 2001; Watts et al. 2025). These signaling cascades may drive transcriptional or metabolic changes that modulate *Dao* and *Aldh1a1* expression. While our study did not directly examine these mechanisms, the results support a model in which microglial VRK2‐dependent TNF‐α signaling modulates astrocytic GABA synthesis. Further studies are needed to clarify the specific molecular mediators involved.

Tonic inhibition, while long considered a passive background signal, is now understood as a dynamic and regionally specialized form of regulation. Dysregulation of tonic GABA has been implicated in various NDDs, including epilepsy, schizophrenia, and ASD (Koh et al. 2023). Depending on the brain region and disease context, both elevated and diminished tonic inhibition can be pathogenic—excessive inhibition may suppress responsiveness, while insufficient inhibition may cause hyperexcitability and disordered network activity (Koh et al. 2023). Our results place *Vrk2*‐deficient mice within the latter category, where impaired astrocytic GABA synthesis leads to diminished tonic inhibition and potentially contributes to altered thalamocortical processing and cognitive dysfunction.

In conclusion, our study identifies VRK2 as a critical regulator of thalamic inhibitory tone through its role in microglia–astrocyte communication. VRK2 supports microglial homeostasis and TNF‐α cytokine output, which in turn sustains astrocytic GABA synthesis via the DAO–ALDH1A1 pathway. This glial–glial interaction ensures adequate tonic inhibition in the MD thalamus, a key hub for cognition and behavior. These findings establish a mechanistic link between VRK2 genetic risk, glial signaling, and thalamocortical circuit dysfunction, offering a glia‐centered framework for understanding and potentially treating VRK2‐associated NDDs.

## Author Contributions

E.C. conceived and supervised the project. Dongsu.L. and E.C. designed experiments and wrote the manuscript. Dongsu.L., G.E.H., Y.L., Denise.L., J.L., and J.H.Y. conducted experiments and performed data analysis. L.C. and H.‐K.K. contributed to sorting thalamic microglia and edited the manuscript. K.W.J. and K.‐T.K. contributed to the generation of *Vrk2*‐deficient mice and edited the manuscript.

## Ethics Statement

All required animal ethics approvals have been obtained for this research.

## Conflicts of Interest

The authors declare no conflicts of interest.

## Supporting information


**Appendix S1:** Supplementary information.

## Data Availability

The data that support the findings of this study are available from the corresponding author upon reasonable request.

## References

[glia70101-bib-0001] Anastasiades, P. G. , D. P. Collins , and A. G. Carter . 2021. “Mediodorsal and Ventromedial Thalamus Engage Distinct L1 Circuits in the Prefrontal Cortex.” Neuron 109, no. 2: 314–330. 10.1016/j.neuron.2020.10.031. 33188733 PMC7855187

[glia70101-bib-0002] Anticevic, A. , K. Haut , J. D. Murray , et al. 2015. “Association of Thalamic Dysconnectivity and Conversion to Psychosis in Youth and Young Adults at Elevated Clinical Risk.” JAMA Psychiatry 72, no. 9: 882–891. 10.1001/jamapsychiatry.2015.0566. 26267151 PMC4892891

[glia70101-bib-0003] Anticevic, A. , G. Yang , A. Savic , et al. 2014. “Mediodorsal and Visual Thalamic Connectivity Differ in Schizophrenia and Bipolar Disorder With and Without Psychosis History.” Schizophrenia Bulletin 40, no. 6: 1227–1243. 10.1093/schbul/sbu100. 25031221 PMC4193728

[glia70101-bib-0004] Bravo‐Ferrer, I. , B. S. Khakh , and B. Diaz‐Castro . 2022. “Cell‐Specific RNA Purification to Study Translatomes of Mouse Central Nervous System.” STAR Protocols 3, no. 2: 101397. 10.1016/j.xpro.2022.101397. 35620074 PMC9127423

[glia70101-bib-0005] Canellada, A. , E. Cano , L. Sanchez‐Ruiloba , F. Zafra , and J. M. Redondo . 2006. “Calcium‐Dependent Expression of TNF‐Alpha in Neural Cells is Mediated by the Calcineurin/NFAT Pathway.” Molecular and Cellular Neuroscience 31, no. 4: 692–701. 10.1016/j.mcn.2005.12.008. 16458016

[glia70101-bib-0006] Chen, P. , E. Ye , X. Jin , Y. Zhu , and L. Wang . 2019. “Association between Thalamocortical Functional Connectivity Abnormalities and Cognitive Deficits in Schizophrenia.” Scientific Reports 9, no. 1: 2952. 10.1038/s41598-019-39367-z. 30814558 PMC6393449

[glia70101-bib-0007] Collins, D. P. , P. G. Anastasiades , J. J. Marlin , and A. G. Carter . 2018. “Reciprocal Circuits Linking the Prefrontal Cortex with Dorsal and Ventral Thalamic Nuclei.” Neuron 98, no. 2: 366eLocator>–379. 10.1016/j.neuron.2018.03.024. 29628187 PMC6422177

[glia70101-bib-0008] Consortium, E. , E. M. Consortium , M. Steffens , et al. 2012. “Genome‐Wide Association Analysis of Genetic Generalized Epilepsies Implicates Susceptibility Loci at 1q43, 2p16.1, 2q22.3 and 17q21.32.” Human Molecular Genetics 21, no. 24: 5359–5372. 10.1093/hmg/dds373. 22949513

[glia70101-bib-0009] Fang, Z. , Y. Dang , A. Ping , et al. 2025. “Human High‐Order Thalamic Nuclei Gate Conscious Perception Through the Thalamofrontal Loop.” Science 388, no. 6742: eadr3675. 10.1126/science.adr3675. 40179184

[glia70101-bib-0010] Ferguson, B. R. , and W. J. Gao . 2018. “Thalamic Control of Cognition and Social Behavior Via Regulation of Gamma‐Aminobutyric Acidergic Signaling and Excitation/Inhibition Balance in the Medial Prefrontal Cortex.” Biological Psychiatry 83, no. 8: 657–669. 10.1016/j.biopsych.2017.11.033. 29373121 PMC5862785

[glia70101-bib-0011] Giraldo‐Chica, M. , B. P. Rogers , S. M. Damon , B. A. Landman , and N. D. Woodward . 2018. “Prefrontal‐Thalamic Anatomical Connectivity and Executive Cognitive Function in Schizophrenia.” Biological Psychiatry 83, no. 6: 509–517. 10.1016/j.biopsych.2017.09.022. 29113642 PMC5809301

[glia70101-bib-0012] Green, T. R. F. , and R. K. Rowe . 2024. “Quantifying Microglial Morphology: An Insight Into Function.” Clinical and Experimental Immunology 216, no. 3: 221–229. 10.1093/cei/uxae023. 38456795 PMC11097915

[glia70101-bib-0013] Griffin, A. L. 2015. “Role of the Thalamic Nucleus Reuniens in Mediating Interactions Between the Hippocampus and Medial Prefrontal Cortex During Spatial Working Memory.” Frontiers in Systems Neuroscience 9: 29. 10.3389/fnsys.2015.00029. 25805977 PMC4354269

[glia70101-bib-0014] Jha, M. K. , M. Jo , J. H. Kim , and K. Suk . 2019. “Microglia‐Astrocyte Crosstalk: An Intimate Molecular Conversation.” Neuroscientist 25, no. 3: 227–240. 10.1177/1073858418783959. 29931997

[glia70101-bib-0015] Ju, Y. H. , M. Bhalla , S. J. Hyeon , et al. 2022. “Astrocytic Urea Cycle Detoxifies Abeta‐Derived Ammonia While Impairing Memory in Alzheimer's Disease.” Cell Metabolism 34, no. 8: 1104–1120. 10.1016/j.cmet.2022.05.011. 35738259

[glia70101-bib-0016] Karimi, B. , P. Silwal , S. Booth , et al. 2021. “Schizophrenia‐Associated LRRTM1 Regulates Cognitive Behavior Through Controlling Synaptic Function in the Mediodorsal Thalamus.” Molecular Psychiatry 26, no. 11: 6912–6925. 10.1038/s41380-021-01146-6. 33981006

[glia70101-bib-0017] Koh, W. , H. Kwak , E. Cheong , and C. J. Lee . 2023. “GABA Tone Regulation and Its Cognitive Functions in the Brain.” Nature Reviews. Neuroscience 24, no. 9: 523–539. 10.1038/s41583-023-00724-7. 37495761

[glia70101-bib-0018] Kwak, H. , W. Koh , S. Kim , et al. 2020. “Astrocytes Control Sensory Acuity via Tonic Inhibition in the Thalamus.” Neuron 108, no. 4: 691–706. 10.1016/j.neuron.2020.08.013. 32905785

[glia70101-bib-0019] Lee, J. , S. Lee , Y. J. Ryu , et al. 2019. “Vaccinia‐Related Kinase 2 Plays a Critical Role in Microglia‐Mediated Synapse Elimination During Neurodevelopment.” Glia 67, no. 9: 1667–1679. 10.1002/glia.23638. 31050055

[glia70101-bib-0020] Lee, J. H. , J. Y. Kim , S. Noh , et al. 2021. “Astrocytes Phagocytose Adult Hippocampal Synapses For Circuit Homeostasis.” Nature 590, no. 7847: 612–617. 10.1038/s41586-020-03060-3. 33361813

[glia70101-bib-0021] Lee, J. U. , L. K. Kim , and J. M. Choi . 2018. “Revisiting the Concept of Targeting NFAT to Control T Cell Immunity and Autoimmune Diseases.” Frontiers in Immunology 9: 2747. 10.3389/fimmu.2018.02747. 30538703 PMC6277705

[glia70101-bib-0022] Lee, S. , T. Ahmed , S. Lee , et al. 2011. “Bidirectional Modulation of Fear Extinction by Mediodorsal Thalamic Firing in Mice.” Nature Neuroscience 15, no. 2: 308–314. 10.1038/nn.2999. 22197828

[glia70101-bib-0023] Li, M. , Y. Wang , X. B. Zheng , et al. 2012. “Meta‐Analysis and Brain Imaging Data Support the Involvement of VRK2 (rs2312147) in Schizophrenia Susceptibility.” Schizophrenia Research 142, no. 1–3: 200–205. 10.1016/j.schres.2012.10.008. 23102693

[glia70101-bib-0024] Li, Q. , and B. A. Barres . 2018. “Microglia and Macrophages in Brain Homeostasis And Disease.” Nature Reviews Immunology 18, no. 4: 225–242. 10.1038/nri.2017.125. 29151590

[glia70101-bib-0025] Li, Y. , X. F. Du , C. S. Liu , Z. L. Wen , and J. L. Du . 2012. “Reciprocal Regulation Between Resting Microglial Dynamics And Neuronal Activity In Vivo.” Developmental Cell 23, no. 6: 1189–1202. 10.1016/j.devcel.2012.10.027. 23201120

[glia70101-bib-0026] Matejuk, A. , and R. M. Ransohoff . 2020. “Crosstalk Between Astrocytes and Microglia: An Overview.” Frontiers in Immunology 11: 1416. 10.3389/fimmu.2020.01416. 32765501 PMC7378357

[glia70101-bib-0027] Matuleviciute, R. , E. T. Akinluyi , T. A. O. Muntslag , et al. 2023. “Microglial Contribution to the Pathology of Neurodevelopmental Disorders in Humans.” Acta Neuropathologica 146, no. 5: 663–683. 10.1007/s00401-023-02629-2. 37656188 PMC10564830

[glia70101-bib-0028] Mitchell, A. S. 2015. “The Mediodorsal Thalamus as a Higher Order Thalamic Relay Nucleus Important for Learning and Decision‐Making.” Neuroscience and Biobehavioral Reviews 54: 76–88. 10.1016/j.neubiorev.2015.03.001. 25757689

[glia70101-bib-0029] Nair, A. , J. M. Treiber , D. K. Shukla , P. Shih , and R. A. Muller . 2013. “Impaired Thalamocortical Connectivity in Autism Spectrum Disorder: A Study of Functional and Anatomical Connectivity.” Brain 136, no. Pt 6: 1942–1955. 10.1093/brain/awt079. 23739917 PMC3673456

[glia70101-bib-0030] Park, J. H. , Y. H. Ju , J. W. Choi , et al. 2019. “Newly Developed Reversible MAO‐B Inhibitor Circumvents the Shortcomings of Irreversible Inhibitors in Alzheimer's Disease.” Science Advances 5, no. 3: eaav0316. 10.1126/sciadv.aav0316. 30906861 PMC6426469

[glia70101-bib-0031] Paydar, A. , B. Lee , G. Gangadharan , S. Lee , E. M. Hwang , and H. S. Shin . 2014. “Extrasynaptic GABAA Receptors in Mediodorsal Thalamic Nucleus Modulate Fear Extinction Learning.” Molecular Brain 7: 39. 10.1186/1756-6606-7-39. 24886120 PMC4066285

[glia70101-bib-0032] Pergola, G. , P. Selvaggi , S. Trizio , A. Bertolino , and G. Blasi . 2015. “The Role of the Thalamus in Schizophrenia From a Neuroimaging Perspective.” Neuroscience and Biobehavioral Reviews 54: 57–75. 10.1016/j.neubiorev.2015.01.013. 25616183

[glia70101-bib-0033] Rubin, R. D. , P. D. Watson , M. C. Duff , and N. J. Cohen . 2014. “The Role of the Hippocampus in Flexible Cognition And Social Behavior.” Frontiers in Human Neuroscience 8: 742. 10.3389/fnhum.2014.00742. 25324753 PMC4179699

[glia70101-bib-0034] Shastri, A. , D. M. Bonifati , and U. Kishore . 2013. “Innate Immunity and Neuroinflammation.” Mediators of Inflammation 2013: 342931. 10.1155/2013/342931. 23843682 PMC3697414

[glia70101-bib-0036] Sherman, S. M. 2007. “The Thalamus is More Than Just a Relay.” Current Opinion in Neurobiology 17, no. 4: 417–422. 10.1016/j.conb.2007.07.003. 17707635 PMC2753250

[glia70101-bib-0035] Sherman, S. M. , and R. W. Guillery . 2011. “Distinct Functions for Direct and Transthalamic Corticocortical Connections.” Journal of Neurophysiology 106, no. 3: 1068–1077. 10.1152/jn.00429.2011. 21676936

[glia70101-bib-0037] Silva, B. A. , C. T. Gross , and J. Graff . 2016. “The Neural Circuits of Innate Fear: Detection, Integration, Action, and Memorization.” Learning Membrane 23, no. 10: 544–555. 10.1101/lm.042812.116. PMC5026211 27634145

[glia70101-bib-0038] Spangenberg, E. , P. L. Severson , L. A. Hohsfield , et al. 2019. “Sustained Microglial Depletion With CSF1R Inhibitor Impairs Parenchymal Plaque Development in an Alzheimer's Disease Model.” Nature Communications 10, no. 1: 3758. 10.1038/s41467-019-11674-z. PMC6704256 31434879

[glia70101-bib-0039] Steinberg, S. , S. de Jong , Irish Schizophrenia Genomics, C , et al. 2011. “Common Variants at VRK2 and TCF4 Conferring Risk of Schizophrenia.” Human Molecular Genetics 20, no. 20: 4076–4081. 10.1093/hmg/ddr325. 21791550 PMC3298077

[glia70101-bib-0040] Tait Wojno, E. D. , C. A. Hunter , and J. S. Stumhofer . 2019. “The Immunobiology of the Interleukin‐12 Family: Room for Discovery.” Immunity 50, no. 4: 851–870. 10.1016/j.immuni.2019.03.011. 30995503 PMC6472917

[glia70101-bib-0041] Tsitsikov, E. N. , D. Laouini , I. F. Dunn , et al. 2001. “TRAF1 is a Negative Regulator of TNF Signaling Enhanced TNF Signaling in TRAF1‐Deficient Mice.” Immunity 15, no. 4: 647–657. 10.1016/s1074-7613(01)00207-2. 11672546

[glia70101-bib-0042] Vazquez‐Cedeira, M. , and P. A. Lazo . 2012. “Human VRK2 (Vaccinia‐Related Kinase 2) Modulates Tumor Cell Invasion by Hyperactivation of NFAT1 and Expression Of Cyclooxygenase‐2.” Journal of Biological Chemistry 287, no. 51: 42739–42750. 10.1074/jbc.M112.404285. 23105117 PMC3522273

[glia70101-bib-0043] Watts, T. H. , K. K. M. Yeung , T. Yu , S. Lee , and R. Eshraghisamani . 2025. “TNF/TNFR Superfamily Members in Costimulation of T Cell Responses‐Revisited.” Annual Review of Immunology 43, no. 1: 113–142. 10.1146/annurev-immunol-082423-040557. 39745933

[glia70101-bib-0044] Woodward, N. D. , H. Karbasforoushan , and S. Heckers . 2012. “Thalamocortical Dysconnectivity in Schizophrenia.” American Journal of Psychiatry 169, no. 10: 1092–1099. 10.1176/appi.ajp.2012.12010056. 23032387 PMC3810300

[glia70101-bib-0045] Yamamoto, M. , M. Kim , H. Imai , Y. Itakura , and G. Ohtsuki . 2019. “Microglia‐Triggered Plasticity of Intrinsic Excitability Modulates Psychomotor Behaviors in Acute Cerebellar Inflammation.” Cell Reports 28, no. 11: 2923–2938. 10.1016/j.celrep.2019.07.078. 31509752

[glia70101-bib-0046] Zelikowsky, M. , S. Hersman , M. K. Chawla , C. A. Barnes , and M. S. Fanselow . 2014. “Neuronal Ensembles in Amygdala, Hippocampus, and Prefrontal Cortex Track Differential Components Of Contextual Fear.” Journal of Neuroscience 34, no. 25: 8462–8466. 10.1523/JNEUROSCI.3624-13.2014. 24948801 PMC4061389

[glia70101-bib-0047] Zhang, B. , C. Y. Gao , H. B. Zhang , et al. 2015. “Association of the VRK2 Gene rs3732136 Polymorphism With Schizophrenia in a Northwest Chinese Han Population.” Genetics and Molecular Research 14, no. 3: 9404–9411. 10.4238/2015.August.14.4. 26345874

[glia70101-bib-0048] Zhou, T. , H. Zhu , Z. Fan , et al. 2017. “History of Winning Remodels Thalamo‐PFC Circuit to Reinforce Social Dominance.” Science 357, no. 6347: 162–168. 10.1126/science.aak9726. 28706064

