# Supplementary material for: Microglial VRK2 Regulates Astrocytic GABA Synthesis and Tonic Inhibition in the Thalamus
Source: Glia. 2025 Nov 20;74(1):e70101. doi: 10.1002/glia.70101 (PMC12631724; doi:10.1002/glia.70101)
Supplement: Supplementary file 1 — Appendix S1: Supplementary information. [file GLIA-74-0-s001.docx]

**Microglial VRK2 regulates astrocytic GABA synthesis and tonic inhibition in the thalamus.**

**Running title:** Microglia shape astrocytic GABA output

Dongsu Lee^1^, Go Eun Ha^1^, Yeleen Lee^1^, Denise Lee^1^, Jongseo Lee^1^, Jae Ho Yoon^1^, Leechung Chang^5^, Kyung Won Jo^2,3^, Ho-Keun Kwon^5,6,7^, Kyong-Tai Kim^2, 4^ and Eunji Cheong^1,*^

^1^ Department of Biotechnology, College of Life Science and Biotechnology, Yonsei University, Seoul, 03722, Republic of Korea

^2^ Laboratory of Molecular Neurophysiology, Department of Life Sciences, Pohang University of Science and Technology (POSTECH), Pohang, Gyeongbuk, 37673, Republic of Korea.

^3^ Present address: Hesed Bio Corporation, Pohang, Gyeongbuk, 37673, Republic of Korea

^4^ Present address: Generative Genomics Research Center, Global Green Research & Development Center, Handong Global University, Pohang, 37554, Republic of Korea.

^5^ Department of Microbiology and Immunology, Yonsei University College of Medicine, Seoul, 03722, Republic of Korea.

^6^ Institute for Immunology and Immunological Diseases, Yonsei University College of Medicine, Seoul, 03722, Republic of Korea.

^7^ Brain Korea 21 PLUS Project for Medical Sciences, Yonsei University College of Medicine, Seoul, 03722, Republic of Korea.

* Corresponding author


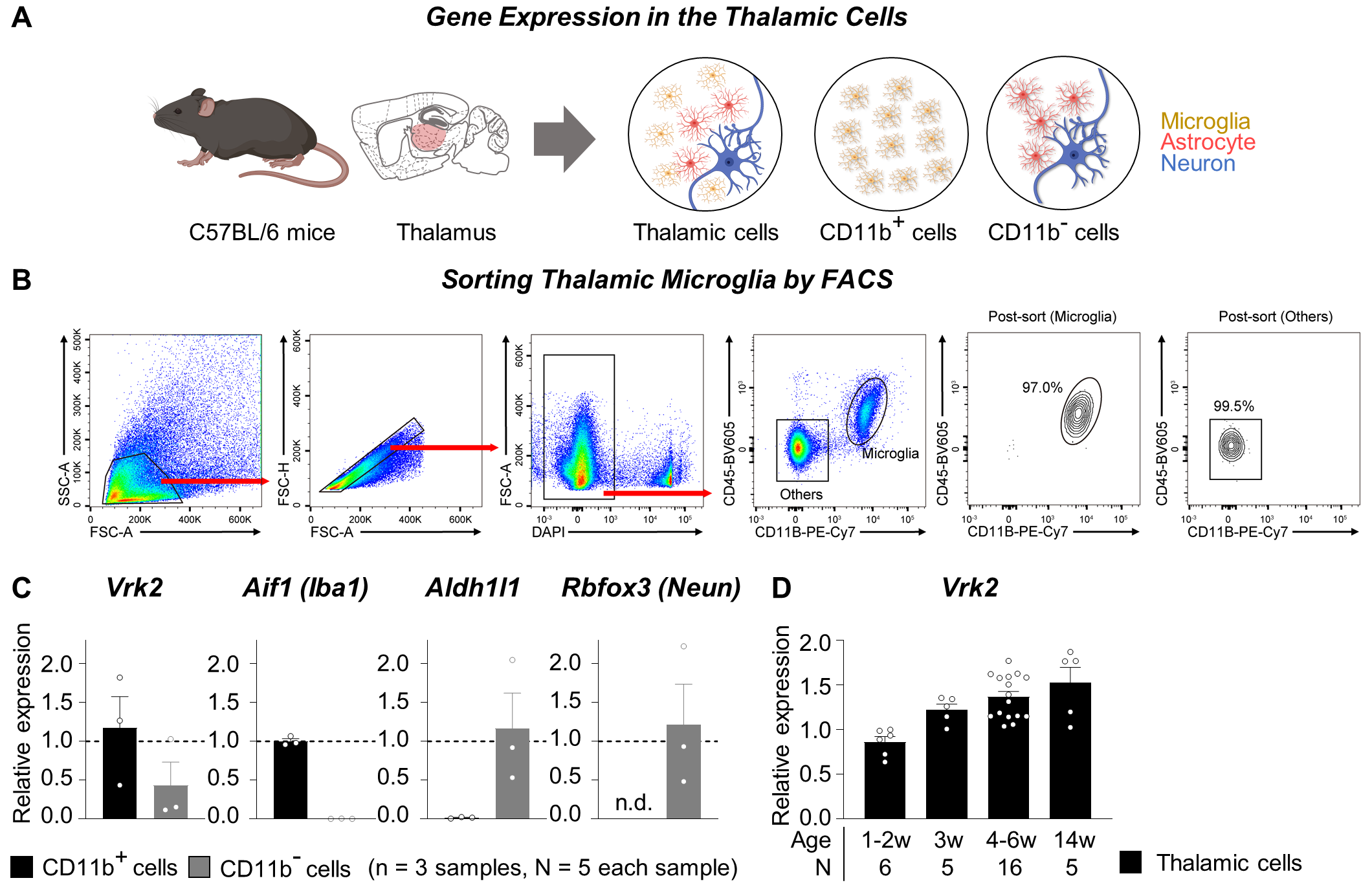


**Figure S1. Validation of *Vrk2* expression in the sorted thalamic microglia and age-dependent *Vrk2* expression in the thalamus.** (**A**) Schematic of quantitative PCR (qPCR) using thalamic cells. (**B**) Representative cytogram of fluorescence-activated cell sorter (FACS), sorting microglia from the thalamic cells using forward scatter (FSC), side scatter (SSC), DAPI, CD45, and CD11b signal. (**C**) Gene expression of *Vrk2*, *Aif1* (or *Iba1*), *Aldh1l1*, and *Rbfox3* (or *Neun*) expression of sorted thalamic cells. (**D**) Age-dependent *Vrk2* expression of the thalamus in the 1-2, 3, 4-6, and 14-week-old mice. Expression in the 4-6 week old mice adapted from **Figure 1B**. ‘n’ denotes the number of samples (**C**) and ‘N’ denotes the number of mice (**C** and **D**). Data are presented as mean ± SEM or not detected (n.d.) (**C** and **D**).


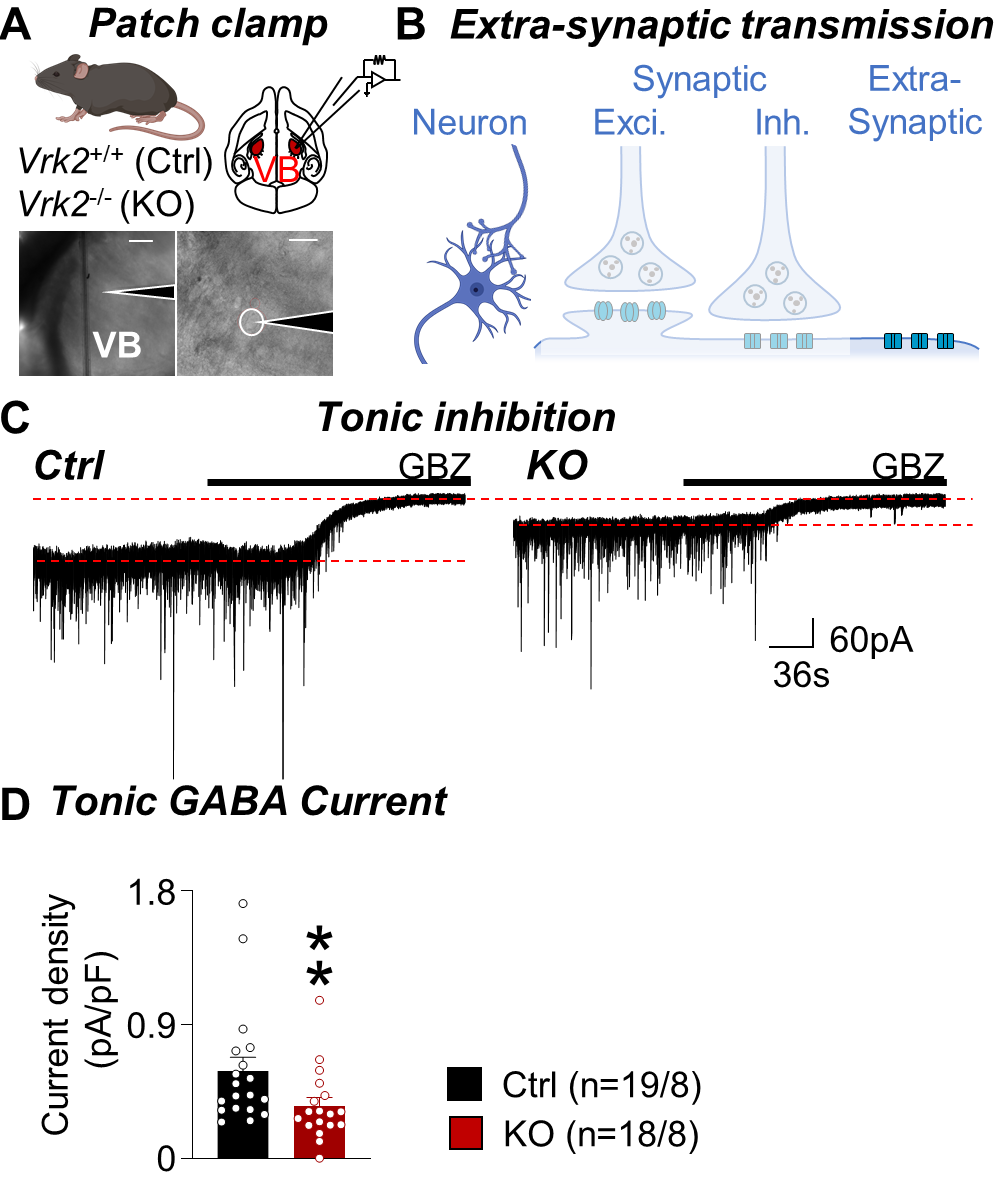


**Figure S2. Tonic GABA current of ventrobasal thalamus in the *Vrk2*-deficient mice.** (**A**) Schematic (*top*) and representative images (*bottom*) of *ex vivo* electrophysiology of the ventrobasal (VB) thalamus in the control (*Vrk2*^+/+^, Ctrl) and *Vrk2*-deficient (*Vrk2*^-/-^, KO) mice. Scale bar, 200 μm and 20 μm. (**B**) Schematic of extra-synaptic transmission in the thalamus. (**C**) Representative trace of tonic inhibition in the VB thalamus in Ctrl and KO mice. (**D**) Quantification of tonic GABA current in the VB thalamus in Ctrl and KO mice. ‘n’ denotes the number of cells/mice. Data are presented as mean ± SEM. ***p* < 0.01, Mann-Whitney test.


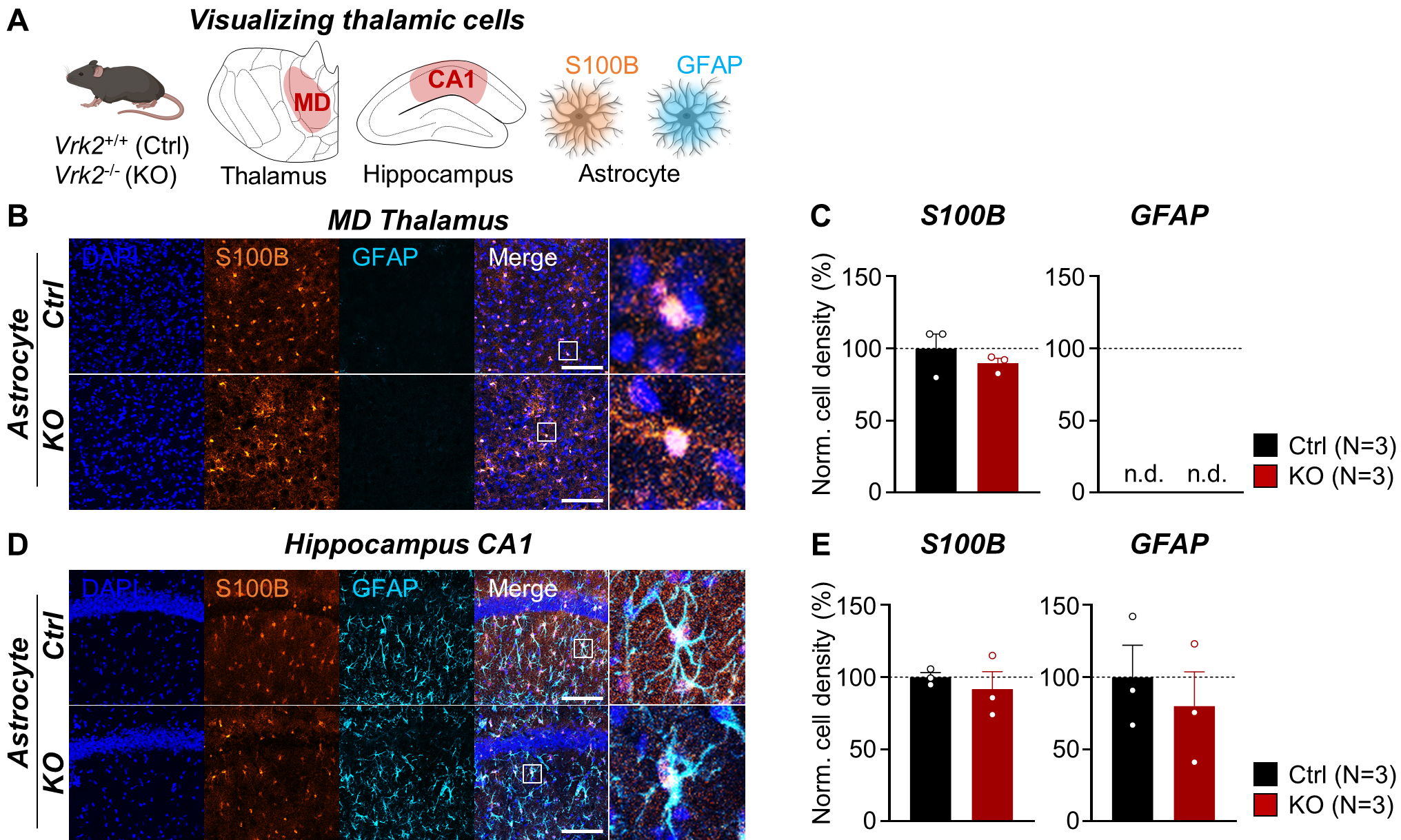


**Figure S3 Cell density of S100B^+^ and GFAP^+^ astrocytes in the *Vrk2*-deficient mice.** (**A**) Schematic of visualizing S100B^+^ and GFAP^+^ astrocytes in the control (*Vrk2*^+/+^, Ctrl) and *Vrk2*-deficient (*Vrk2*^-/-^, KO) mice. (**B**) Representative images for S100B (orange) and GFAP (cyan) with DAPI in the mediodorsal (MD) thalamus of *Vrk2*-deficient mice. Scale bar 100 μm. (**C**) Quantification of the relative proportion of cell density of S100B^+^ and GFAP^+^ cells in the MD thalamus of *Vrk2*-deficient mice. (**D**) Representative images for S100B (orange) and GFAP (cyan) with DAPI in the hippocampal CA1 of *Vrk2*-deficient mice. Scale bar 100 μm. (**E**) Quantification of the relative proportion of cell density of S100B^+^ and GFAP^+^ cells in the hippocampal CA1 of *Vrk2*-deficient mice. ’N’ denotes the number of mice (**C** and **E**). Data are presented as mean ± SEM or not detected (n.d.) (**C** and **E**).


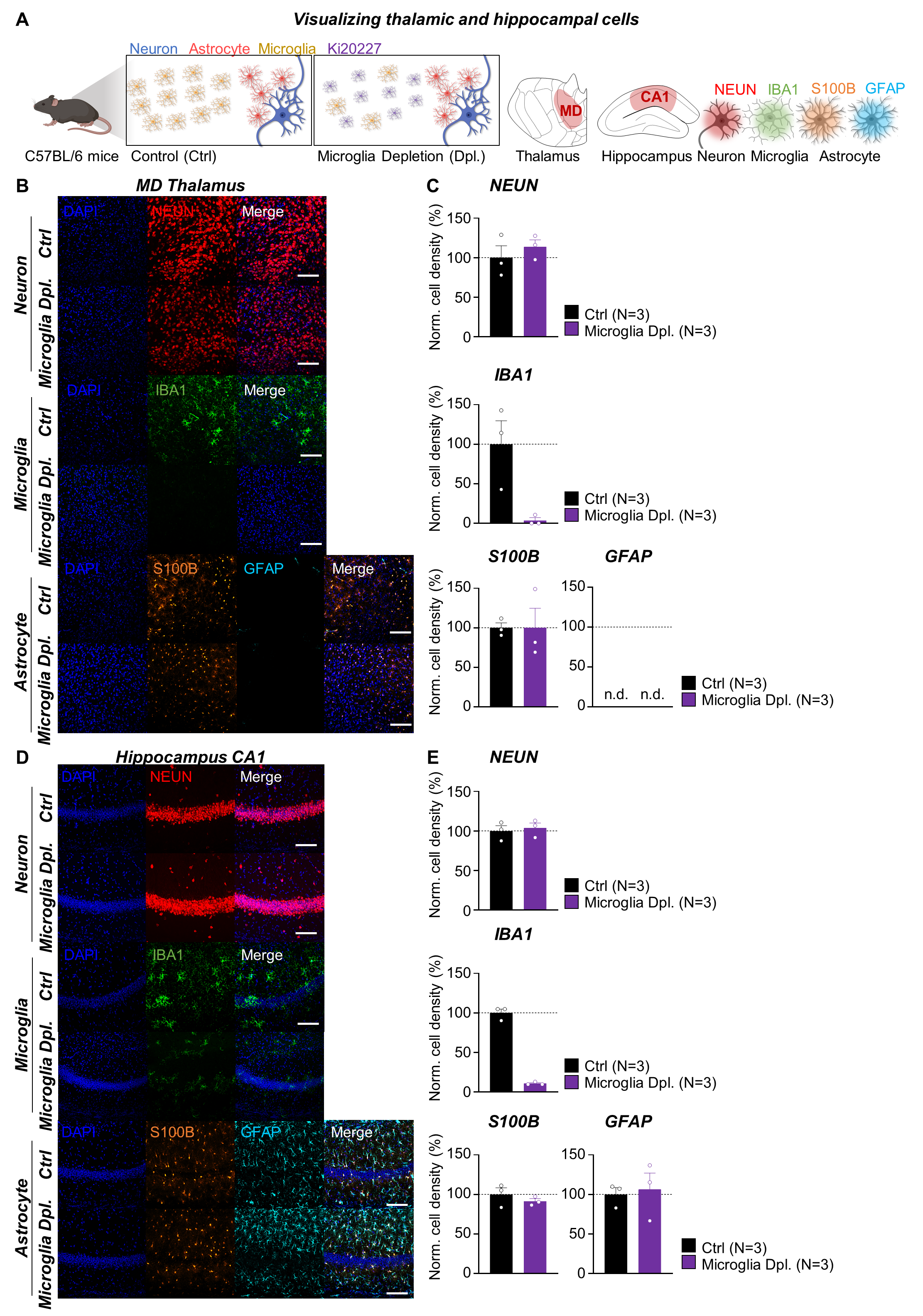


**Figure S4.Cell density of neurons, microglia, and astrocytes in Ki20227-administered mice.** (**A**) Schematic of visualizing neurons, microglia, and astrocytes in the control (Ctrl) and microglia depletion (microglia Dpl.) condition by Ki20227-administration. (**B**) Representative images for NEUN (red, *top*), IBA1 (green, *middle*), S100B (orange, *bottom*), and GFAP (cyan, *bottom*) in the mediodorsal (MD) thalamus of Ki20227-administered mice. Scale bar 100 μm. (**C**) Quantification of the relative proportion of cell density of NEUN^+^ (*top*), IBA1^+^ (*middle*), S100B^+^ (*bottom*), and GFAP^+^ (*bottom*) cells in the MD thalamus of Ctrl and microglia Dpl. condition. (**D**) Representative images for NEUN (red, *top*), IBA1 (green, *middle*), S100B (orange, *bottom*), and GFAP (cyan, *bottom*) in the hippocampus CA1 of Ki20227-administered mice. (**E**) Quantification of the relative proportion of cell density of NEUN^+^ (*top*), IBA1^+^ (*middle*), S100B^+^ (*bottom*), and GFAP^+^ (*bottom*) cells in the hippocampus CA1 of Ctrl and microglia Dpl. condition. ’N’ denotes the number of mice (**C** and **E**). Data are presented as mean ± SEM or not detected (n.d.) (**C** and **E**).


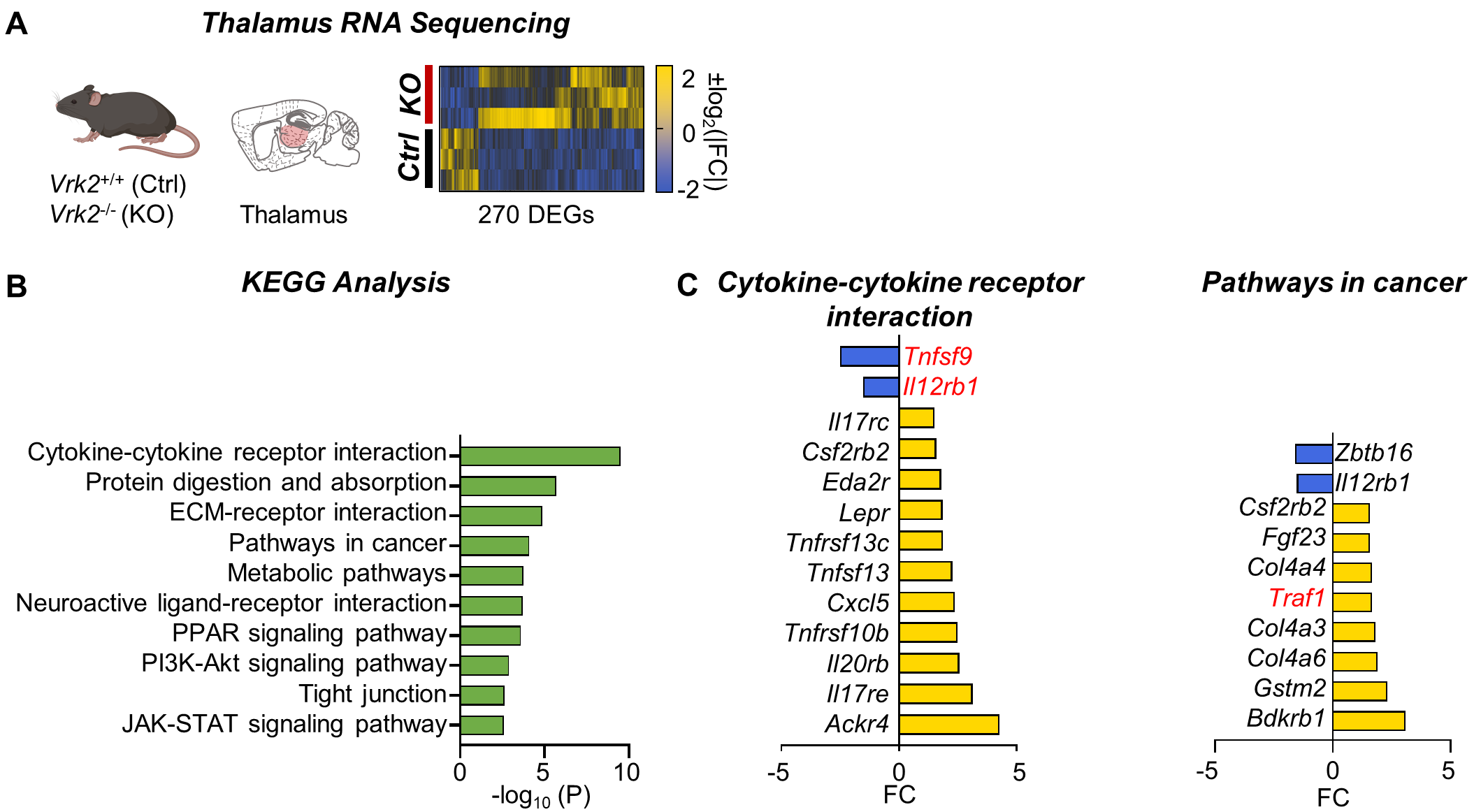


**Figure S5. The molecular identity of the thalamic cells in the *Vrk2*-deficient mice.** (**A**) Schematic (*left*) and heat map (*right*) of gene expression of the thalamus in the control (*Vrk2*^+/+^, Ctrl) and *Vrk2*-deficient (*Vrk2*^-/-^, KO) mice. Adapted from **Figure 3A**. (**B**) Quantification of -log_10 (_p-value) [-log_10_(P)] of gene expression in the top 10 KEGG pathways. Adapted from **Figure 3D**. (**C**) Quantification of fold change (FC) for each gene in KEGG pathways; Cytokine-cytokine receptor interaction; *Tnfsf9*, *Il12rb1*, *Il17rc*, *Csf2rb2*, *Eda2r*, *Lepr*, *Tnfrsf13c*, *Tnfsf13*, *Cxcl5*, *Tnfrsf10b*, *Il20rb*, *Il17re*, and *Ackr4*; Pathways in cancer; *Zbtb16*, *Il12rb1*, *Csf2rb2*, *Fgf23*, *Col4a4*, *Traf1*, *Col4a3*, *Col4a6*, *Gstm2*, and *Bdkrb1*. Data are presented as –log_10_(P) (**B**) and FC (**C**).


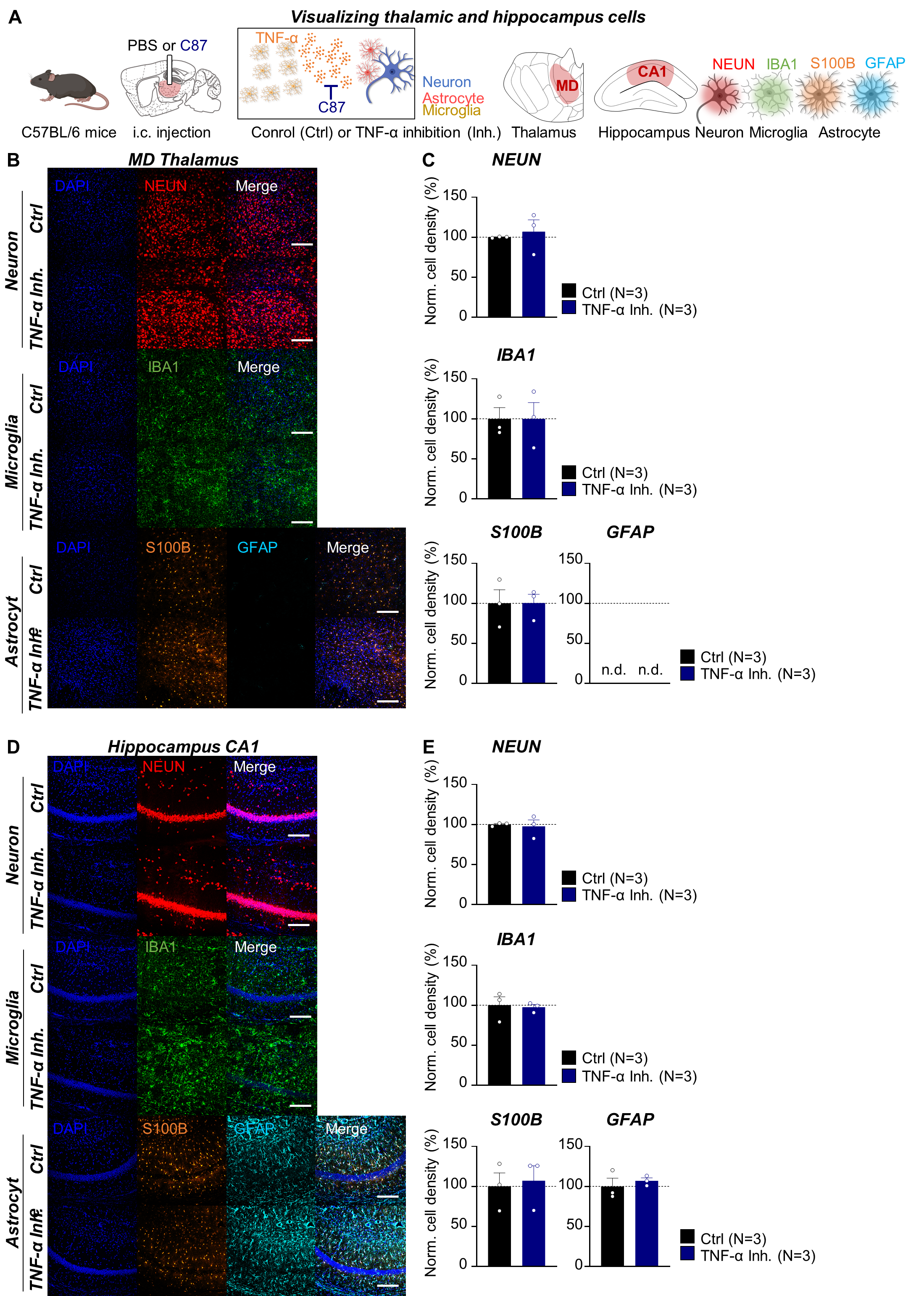


**Figure S6.Cell density of neurons, microglia, and astrocytes in the C87-injected mice.** (**A**) Schematic of visualizing neurons, microglia, and astrocytes in the control (Ctrl) and TNF-α inhibition (TNF-α Inh.) condition by C87-injection. (**B**) Representative images for NEUN (red, *top*), IBA1 (green, *middle*), S100B (orange, *bottom*), and GFAP (cyan, *bottom*) in the mediodorsal (MD) thalamus of Ctrl and TNF-α Inh. condition. Scale bar 100 μm. (**C**) Quantification of the relative proportion of cell density of NEUN^+^ (*top*), IBA1^+^ (*middle*), S100B^+^ (*bottom*), and GFAP^+^ (*bottom*) cells in the MD thalamus of Ctrl and TNF-α Inh. condition. (**D**) Representative images for NEUN (red, *top*), IBA1 (green, *middle*), S100B (orange, *bottom*), and GFAP (cyan, *bottom*) in the hippocampus CA1 of Ctrl and TNF-α Inh. condition. (**E**) Quantification of the relative proportion of cell density of NEUN^+^ (*top*), IBA1^+^ (*middle*), S100B^+^ (*bottom*), and GFAP^+^ (*bottom*) cells in the hippocampus CA1 of Ctrl and TNF-α Inh. condition. ’N’ denotes the number of mice (**C** and **E**). Data are presented as mean ± SEM or not detected (n.d.) (**C** and **E**).
